# Combined single-cell profiling of chromatin–transcriptome and splicing across brain cell types, regions and disease state

**DOI:** 10.1038/s41587-025-02734-5

**Published:** 2025-07-22

**Authors:** Wen Hu, Careen Foord, Justine Hsu, Li Fan, Michael J. Corley, Samantha N. Lanjewar, Siwei Xu, Natan Belchikov, Yi He, Alina P. S. Pang, Tarun N. Bhatia, Julien Jarroux, Anoushka Joglekar, Teresa A. Milner, Lishomwa C. Ndhlovu, Jing Zhang, Eduardo Butelman, Steven A. Sloan, Virginia M. Y. Lee, Li Gan, Hagen U. Tilgner

**Affiliations:** 1 Feil Family Brain and Mind Research Institute, Weill Cornell Medicine, New York, NY USA; 2 Center for Neurogenetics, Weill Cornell Medicine, New York, NY USA; 3 Helen and Robert Appel Alzheimer’s Disease Research Institute, New York, NY USA; 4 Department of Medicine, Division of Infectious Diseases, Weill Cornell Medicine, New York, NY USA; 5 Department of Human Genetics, Emory University School of Medicine, Atlanta, GA USA; 6 Department of Computer Science, University of California, Irvine, CA USA; 7 Physiology, Biophysics & Systems Biology Program, Weill Cornell Medicine, New York, NY USA; 8 Neuropsychoimaging of Addiction and Related Conditions Research Program, Department of Psychiatry, Icahn School of Medicine at Mount Sinai, New York, NY USA; 9 Center for Neurodegenerative Disease Research, University of Pennsylvania School of Medicine, Philadelphia, PA USA

**Keywords:** Transcriptomics, RNA splicing, Genetics of the nervous system, Next-generation sequencing, Epigenomics

## Abstract

Measuring splicing and chromatin accessibility simultaneously in frozen tissues remains challenging. Here we combined single-cell isoform RNA sequencing and assay for transposase accessible chromatin (ScISOr–ATAC) to interrogate the correlation between these modalities in single cells in human and rhesus macaque frozen cortical tissue samples. Applying a previous definition of four ‘cell states’ in which the transcriptome and chromatin accessibility are coupled or decoupled for each gene, we demonstrate that splicing patterns in one cell state can differ from those of another state within the same cell type. We also use ScISOr–ATAC to measure the correlation of chromatin and splicing across brain cell types, cortical regions and species (macaque and human) and in Alzheimer’s disease. In macaques, some excitatory neuron subtypes show brain-region-specific splicing and chromatin accessibility. In human and macaque prefrontal cortex, strong evolutionary divergence in one molecular modality does not necessarily imply strong divergence in another modality. Finally, in Alzheimer’s disease, oligodendrocytes show high dysregulation in both chromatin and splicing.

## Abstract (web summary)

Joint profiling of chromatin and splicing in the brain uncovers shared and distinct patterns.

## Main

Multimodal measurements, including the simultaneous measurements of gene expression, chromatin accessibility^1–3^ and antibody binding in single-cell^4^ and spatial genomics^5,6^ experiments, are of high importance in neurobiological investigations and modern-day genomics. We have previously devised methods to sequence full-length transcripts, alternative exons and exon combinations in single-cell and single-nuclei studies^7–9^. Here, we introduced chromatin accessibility as an additional modality to observe splicing and chromatin accessibility (assay for transposase accessible chromatin (ATAC)) simultaneously. Moreover, gene expression and ATAC have been used to define a gene’s ‘cell state’, defined as states where transcription (induction and repression) and chromatin (opening and closing) are coupled or decoupled^10^. However, whether such cell states can result in distinct splicing regulation remains unexplored. A recent study showed that genes can exist in distinct states based on transcriptional activity and chromatin accessibility, defined as priming, coupled-on, decoupled and coupled-off (corresponding to cell states 0, 1, 2 and 3). Li et al. defined these states as follows. Priming marks chromatin opening before transcription begins, coupled-on reflects active transcription coupled with open chromatin, decoupling marks the end of transcription, when chromatin closing and transcriptional repression are out of sync, and coupled-off indicates inactive transcription and closed chromatin^10^. We applied this ‘cell state’ framework to identify cell-type-specific splicing changes by cell state.

Both splicing and chromatin organization distinguish cell types within a brain region and across brain regions^7,9,11–14^. Moreover, multiple modalities have undergone evolutionary changes and are affected in complex diseases including Alzheimer’s disease (AD)^15–17^. A key question is whether chromatin and splicing alterations reflect the same underlying processes.

The brain is divided into interconnected regions that are disproportionately affected by distinct neurological diseases. The prefrontal cortex (PFC) is involved in executive and cognitive function, whereas the visual cortex involves visual inputs^18,19^. The PFC is known to be affected in frontotemporal dementia, AD and psychiatric disorders, whereas the visual cortex is affected in cerebral visual impairment^20–26^. These differences highlight the need to understand brain-region-specific molecular features. Macaques (*Macaca mulatta*), although widely used as models due to their evolutionary proximity to humans^27^, may not fully replicate human cell-type-specific molecular patterns. Therefore, detailed analyses of species-specific splicing and chromatin alterations across cell types is essential to assess the reliability of model organisms compared to humans. Last, both splicing and chromatin changes have been linked to AD. Although splicing data mostly come from bulk tissue^16^, single-cell chromatin alterations have been studied. However, it remains unclear whether cell types are equally affected in AD-specific splicing and if the most-affected cell types change between modalities.

**Fig. 1:**
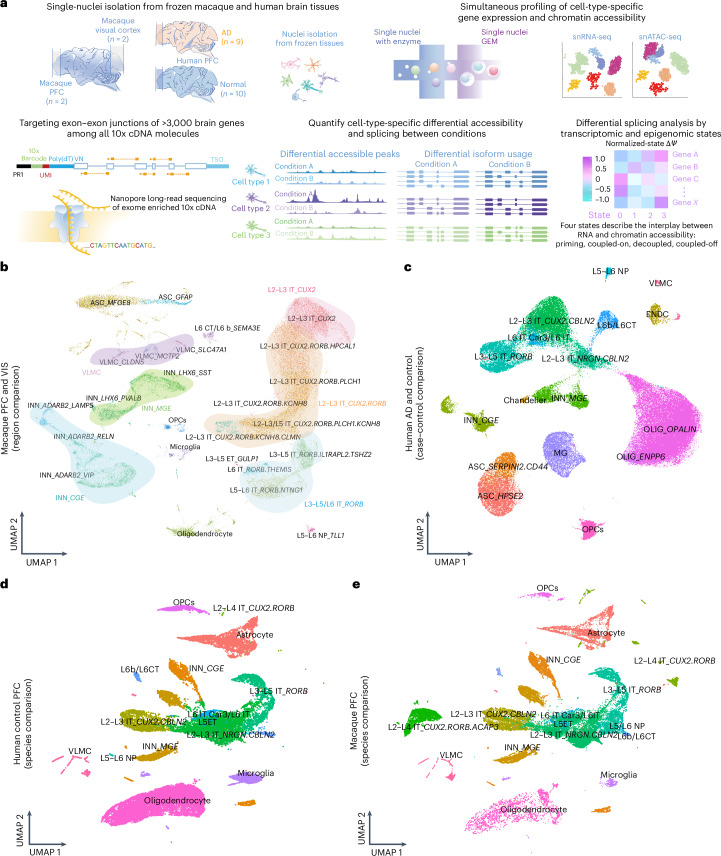
ScISOr-ATAC pipeline and data overview. **a**, Outline of ScISOr–ATAC experimental and analysis pipeline; GEM, Gel Bead-In Emulsion; snRNA-seq, single-nucleus RNA sequencing; snATAC-seq, single-nucleus ATAC with sequencing; TSO, template switch oligo; poly(dT)VN, poly-dT primer sequence. **b**, Uniform manifold approximation and projection (UMAP) of macaque PFC and visual cortex (VIS) samples; ASC, astrocytes; INN, inhibitory neurons; VLMC, vascular and leptomeningeal cells; MG, microglia; OLIG, oligodendrocytes; OPCs, oligodendrocyte precursor cells; ENDC, endothelial cell. Excitatory neurons are indicated by L, IT or ET and gene markers. **c**, UMAP of human AD and control PFC samples. **d**, UMAP of human nuclei from integrated control human PFC and macaque samples. **e**, UMAP of macaque nuclei from integrated control human PFC and macaque samples.

Therefore, we devised single-cell isoform RNA sequencing coupled with ATAC (ScISOr–ATAC), which measures gene expression, splicing and chromatin accessibility in the same individual cells. We used this method to show that distinct cell states (chromatin–transcriptome coupling/decoupling states) can reveal distinct splicing patterns. We then applied ScISOr–ATAC to the macaque PFC and visual cortex, macaque and human PFC and AD diseased and control PFC (Fig. 1a). To circumvent differences in statistical power between cell types, we developed downsampling software that compares statistically equal changes between cell types or conditions (Methods and Code availability).

We consider multiple cell subtypes, especially subtypes of excitatory neurons. We denote excitatory neurons by cortical layer (L), intratelencephalic (IT)/extratelencephalic (ET), corticothalamic (CT) and near-projecting (NP) categories and gene markers. In macaques, we identified three main excitatory subtypes based on layer-specific marker expression of *CUX2*, *RORB* and both, together with other cortical neuron markers (Methods and Supplementary Fig. 1), termed L2–L3 IT_*CUX2*, L3–L5/L6 IT_*RORB* or L2–L4 IT_*CUX2.RORB*. Neuronal subtypes are transcriptionally distinct with unique synaptic properties^28–32^. In mice, *CUX2* marks upper-layer neurons and regulates synaptic functions^33,34^, whereas *RORB* is highly expressed in L4 neurons and is essential for synaptic and chromatin organization^35^.

In brain region comparisons, L3–L5/L6 IT_*RORB* neurons show the strongest splicing specificity, whereas L2–L4 IT_*CUX2.RORB* cells show the highest chromatin specificity. Between macaque and human PFC, chromatin and splicing often affect different cell types. In AD, glial cells show stronger dysregulation than neurons across both modalities. Moreover, exon inclusion varies with the chromatin–transcription cell state, which suggests that these states should be considered as a hidden variable in the analyses. In summary, chromatin and splicing show distinct contributions to within-species brain region specificity, species divergence and AD dysregulation, among distinct cell types, subtypes and chromatin–transcription cell states; however, in specific comparisons, both modalities can agree.

## Results

### Definition of cell types

From two adult male rhesus macaques (Methods), we collected PFC and visual cortex samples guided by the Allen Brain Atlas^36^. Using a 10x Genomics Multiome kit, we prepared single-nucleus RNA and ATAC libraries and sequenced 293 million–385 million paired-end reads for RNA and 350 million–381 million reads for ATAC (Supplementary Fig. 1a). After downsampling reads to similar read numbers per cell and analyzing the RNA data using published tools^37–39^, we identified 36 cell types and subtypes, including astrocytes, oligodendrocytes, oligodendrocyte precursor cells, microglia, endothelial cells and various subtypes of excitatory and inhibitory neurons (Methods, Supplementary Fig. 1b and Supplementary Table 1). Overall, we found 6,858–13,710 cells per sample after filtering, with excitatory neurons being the most abundant (Supplementary Fig. 1c). Within the excitatory neurons, three subtypes stood out: (1) L3–L5/L6 IT_*RORB* neurons, mainly characterized by *RORB* expression along with *IL1RAPL* and *MKX*; (2) L2–L3 IT_*CUX2* neurons, marked by *CUX2*, *HPCAL1* and *CBLN2*; and (3) L2–L4 IT_*CUX2.RORB* neurons, which coexpress both *RORB* and *CUX2* (Fig. 1b and Supplementary Fig. 2a). In primates, *RORB* excitatory neurons reside in layers L3–L5/L6, *CUX2.RORB* excitatory neurons reside in layers L2–L4 and *CUX2* excitatory neurons reside in layers L2–L3 (refs. ^40–44^). Average numbers of RNA and ATAC unique molecular identifiers (UMIs) per cell type between PFC and visual cortex samples (Supplementary Figs. 1f,g and 2b,c) were correlated (Supplementary Figs. 1d,e and 2d,e). Analysis of ten healthy and nine AD-affected human PFC samples (Supplementary Table 2 and Methods) revealed expected brain cell types (Fig. 1c) and largely matched those in macaques. However, two excitatory neuron clusters coexpressing *CUX2* and *RORB* (L2–L4 IT_*CUX2.RORB* and L2–L4 IT_*CUX2.RORB.ACAP3*) were rare in human samples (Fig. 1d,e), potentially due to species differences or sampling bias^45^.

Overall, excitatory neurons were highly abundant across brain regions and species (Fig. 1d,e). To gain insight into disease and synaptic processes, we custom designed an Agilent enrichment array covering all annotated splice junctions in 3,224 macaque and 3,630 human genes (Methods). These consist of genes linked to synaptic function^46^, AD^16^, *TDP43* knockdown^47^, autism spectrum disorder (ASD)^48–50^, schizophrenia^51^ and amyotrophic lateral sclerosis (ALS)^52^ and genes with cell-type-specific splicing patterns in our human PFC^8^ data (Supplementary Fig. 3a,b). We applied this enrichment array to the 10x cDNA for Oxford Nanopore Technologies (ONT) long-read sequencing (Supplementary Fig. 4). We achieved 79% to 83% on-target capture using the enrichment panel, compared to ~2% for the unenriched Illumina reads after in silico extension to the average ONT read length (Supplementary Fig. 4a). This extension artificially expands the mapped Illumina reads to the average ONT read length, enabling fair comparisons of equal length. Conservative calling of barcodes yielded 20 million–33 million perfectly matching barcoded reads per sample (Supplementary Fig. 4b). Reads were mapped to the macaque genome using minimap2 (ref. ^53^) and assigned to genes using scisorseqr^9^. We filtered spliced reads from the mapped and barcoded reads (Supplementary Fig. 4c). Spliced ONT reads mapping to the same gene were considered distinct UMIs if their edit distance was ≥4 (Methods and Supplementary Fig. 4d). ONT read lengths showed similar distributions with a median of 713 bp (Supplementary Fig. 4e). The median of long-read UMI counts varied by cell type, where the lowest was observed in oligodendrocytes (Supplementary Fig. 4f,g), whereas the three main excitatory neuron subtypes (L2–L3 IT_*CUX2*, L2–L4 IT_*CUX2.RORB* and L3–L5/L6 IT_*RORB*) showed similar UMI distributions (Supplementary Fig. 4h,i). The exon junction targeting before long-read sequencing removes purely intronic reads as we have shown before^8^. Moreover, exon-overlapping short-read and long-read UMI counts showed correlations between 0.74 and 0.77 per dataset. This suggests that the targeting process is not drastically biased to certain exons (Supplementary Fig. 4j).

### Region-specific splicing patterns are distinct from chromatin

**Fig. 2:**
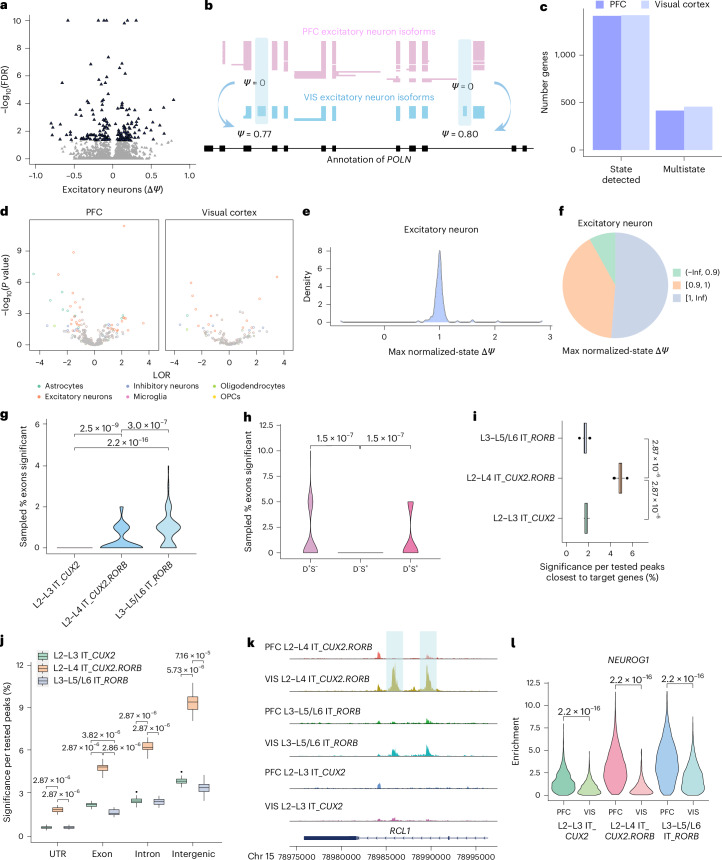
Region specific splicing patterns are distinct from chromatin. **a**, Volcano plot of brain-region-specific splicing for excitatory neurons. **b**, Cell-type-resolved single-cell long reads for *POLN*. Each line represents a single cDNA molecule. The two top tracks represent excitatory neurons in the PFC and visual cortex. The bottom black track shows chromosome (chr) 5: 2190541–2265209. **c**, Number of genes that include exons tested with one or more and two or more cell states detected in PFC and visual cortex samples. **d**, Volcano plot of state-specific exons across multiple cell types in the PFC and visual cortex (only exons with ten or more reads in two or more states were tested and are shown; *n* = 382,108). Exons with a *P* value of ≤0.05 and | LOR | of >1 are labeled in color, whereas all others are in gray; LOR, log odds ratio. A one-sided *χ*^2^ test followed by a Benjamini–Yekutieli multiple testing correction was applied to evaluate the significance of the splicing–cell state association (Methods). **e**, Distribution of the maximum normalized-state Δ*Ψ* per exon. Normalized-state Δ*Ψ* = state Δ*Ψ* /overall Δ*Ψ*. **f**, Pie chart showing the maximum normalized-state Δ*Ψ* split by value into three groups: <0.9, between 0.9 and 1 or ≥1; Inf, infinity. **g**, Downsampling experiment. Distribution of the percentage of exons significant in brain region comparisons per subtype (Methods; *n* = 100). **h**, Downsampling experiment. Distribution of the percentage of exons significantly targeted by disease probes (D^+^S^−^), synaptic probes (D^−^S^+^) or overlapping (D^+^S^+^; Methods; *n* = 100). **i**, Downsampling experiment. The percentage of peaks that are significantly different for each excitatory neuron subtype between brain regions in the vicinity of genes targeted for splicing analysis is shown (Methods; *n* = 20). **j**, Breakdown of the percentage of significant peaks by peak location (UTR, exon, intron or intergenic; Methods; *n* = 20). **k**, Example peaks (shaded areas) in the vicinity of *RCL1* is specific to the visual cortex only in L2–L4 IT_*CUX2.RORB* excitatory neurons. **l**, Motif enrichment of the transcriptional regulator *NEUROG1* for excitatory neuron subtypes in the PFC and visual cortex. Each box plot shows the median (middle line), interquartile range (top and bottom lines of the box) and adjacent values (whiskers extending to 1.5× the interquartile range (IQR)). Dots represent outliers beyond this range. A two-sided Wilcoxon rank-sum test was applied to all the comparisons shown in **g**, **i**, **j** and **l**. FDR correction was applied to multiple comparisons, and corrected *P* values (<0.05) are shown.

Differential gene expression analysis between PFC and visual cortex revealed stronger changes in RNA splicing-related genes in excitatory neurons than in inhibitory neurons (Methods and Supplementary Fig. 5). Given their cortical importance and abundance, we tested 4,818 exons for differential exon inclusion (Δpercent spliced in (Δ*Ψ*)) in excitatory neurons using 2 × 2 exon tests^8,9,54^ and a Benjamini–Yekutieli (false discovery rate (FDR)) correction^55^. We identified 143 significant exons (FDR < 0.05, | Δ*Ψ* | ≥ 0.1; median | Δ*Ψ* | = 0.21; Fig. 2a). Among them, the gene encoding DNA polymerase nu (*POLN*) showed brain-region-specific splicing: two exons are completely skipped in the PFC but are robustly included in the visual cortex (Δ*Ψ* = 0.78 and 0.8; adjusted two-sided Fisher’s exact test *P* values of <0.006 and <0.003) and follow the paradigm of coordinated splicing^7,56–60^ (Fig. 2b). Given that we observed that *POLN* is highly expressed in excitatory neurons (Supplementary Fig. 6a), we validated its two alternative exons using bulk tissue from three macaque PFC and visual cortex samples and observed a broadly similar trend in the tested alternative exons, but not in constitutive exons (Supplementary Fig. 6b,c). Among nonsignificant 2 × 2 tests, 71.7% had a | Δ*Ψ* | of ≤0.1 and 91.1% had a | Δ*Ψ* | of ≤0.2, with very few passing a | Δ*Ψ* | of 0.3, suggesting that most would remain nonsignificant even with deeper sequencing (Supplementary Fig. 6d). However, among the few with a | Δ*Ψ* | of ≥0.5 and 10–20 informative reads in each condition (*n* = 6), many may achieve significance with higher depth. Indeed, simulation experiments suggested that for exons with a | Δ*Ψ* | of ≥0.5, only 38% reached significance if 10–20 reads were sampled (Supplementary Fig. 6e and Methods).

Our data offer the unique opportunity to test whether different cell states^10^ show differences in splicing. We examined exons of genes detectable in multiple states with sufficient long-read coverage in the PFC or visual cortex (Fig. 2c and Methods). Across both brain regions and multiple cell types, we found exons with inclusion differences tied to distinct cell states (Fig. 2d), suggesting a link to the interplay between chromatin and splicing^61,62^. This observation highlights the question of whether observed exon inclusion differences between visual cortex and PFC excitatory neurons reflect cell-state diversities. To test this hypothesis, we analyzed 160 exons with five or more long reads in at least one cell state and brain region for excitatory neurons. For each exon, we calculated a ‘normalized-state Δ*Ψ*’ by dividing the state Δ*Ψ* by the Δ*Ψ* across all states, which we refer to as the ‘overall Δ*Ψ*’. Values of ≥1 indicate that a specific state equaled or exceeded the overall splicing difference. Many exons showed at least one state with a normalized-state Δ*Ψ* of ≥1. In some cases, an exon’s maximum normalized-state Δ*Ψ* values exceeded 1.5, suggesting that strong brain region specificity originates from one state above others (Fig. 2e). In excitatory neurons, 51% of exons (82/160) showed brain-region-specific splicing in at least one cell state, whereas another 41% (65/160) had maximum normalized-state Δ*Ψ* values between 0.9 and 1. The remaining cases likely stemmed from cell state rather than brain region specificity (Fig. 2f).

To assess how excitatory neuron subtypes contribute to region-specific splicing, we compared matched subtypes between the PFC and visual cortex. In L3–L5/L6 IT_*RORB* excitatory neurons, 64 of 1,558 exons showed significant splicing differences (FDR < 0.05, | Δ*Ψ* | ≥ 0.1; median | Δ*Ψ* | = 0.34; Supplementary Table 3 and Supplementary Fig. 7a,b). In L2–L4 IT_*CUX2.RORB* neurons, a higher number of significant exons with a | Δ*Ψ* | of ≥0.1 was found (*n* = 93 of 2,881; Supplementary Table 4 and Supplementary Fig. 7a,b), whereas fewer were found in L2–L3 IT_*CUX2* neurons (*n* = 36 of 1,336 tested; Supplementary Table 5 and Supplementary Fig. 7a,b). After allowing at most five significant exons per gene, 67.1% of differentially included exons (49 of 73) showed a bias toward visual cortex-specific inclusion in L2–L4 IT_*CUX2.RORB* neurons, where negative Δ*Ψ* values correspond to higher inclusion in the visual cortex. By contrast, L3–L5/L6 IT_*RORB* excitatory neurons showed a much more even distribution (two-sided Wilcoxon rank-sum test, *P* < 0.05; Supplementary Fig. 7c). The three subtypes offered distinct statistical power in numbers of exons, cells and reads. We therefore performed downsampling analysis and confirmed that L3–L5/L6 IT_*RORB* neurons showed the strongest brain-region-specific splicing regulation, followed by L2–L4 IT_*CUX2.RORB* neurons (corrected two-sided Wilcoxon rank-sum test *P* values of <2.2 × 10^−16^ (L3–L5/L6 IT_*RORB* versus L2–L3 IT_*CUX2*) and <3 × 10^−7^ (L3–L5/L6 IT_*RORB* versus L2–L4 IT_*CUX2.RORB*); Fig. 2g and Methods). An example of brain-region-specific splicing in L3–L5/L6 IT_*RORB* neurons is an exon of the gene encoding NFE2-like BZIP transcription factor 1 (*NFE2L1*)^59^, which is skipped in the PFC but included in 73% of the visual cortex (corrected two-sided Fisher’s exact test *P* < 0.003; Supplementary Fig. 7d). *NFE2L1* was targeted because of its involvement in ALS and ASD; however, we also target synaptic genes. In total, 46.1% of targeted synaptic genes were also classified as disease-associated splicing-dysregulation genes. To assess whether specific gene categories show brain-region-dependent splicing in L3–L5/L6 IT_*RORB* neurons, we classified targeted genes into three groups: disease associated but not synaptic (D^+^S^−^), synaptic but not disease associated (D^−^S^+^) and both synaptic and disease associated (D^+^S^+^). Downsampling experiments (Methods) showed that D^+^S^−^ genes displayed stronger brain-region-specific splicing patterns than D^−^S^+^ genes (corrected two-sided Wilcoxon rank-sum test *P* < 1.5 × 10^−7^). D^+^S^+^ genes also showed such brain region specificity compared to D^−^S^+^ genes (D^+^S^+^ versus D^−^S^+^, corrected two-sided Wilcoxon rank-sum test *P* < 1.5 × 10^−7^), similar to disease-associated genes in brain-region-specific splicing among L3–L5/L6 IT_*RORB* neurons (Fig. 2h). These findings suggest that splicing differences among excitatory neuron subtypes contribute to functional distinctions between the PFC and visual cortex. Additionally, splicing of disease genes may play a more important role in this distinction than synaptic genes, perhaps indicating that such disease-associated genes are mostly altered in specific brain areas.

Like the RNA analysis described above, the statistical power to detect differential chromatin arrangements can vary between cell types. To guarantee similar statistical power across samples, we randomly subset one experiment so that all four samples had 7,000–8,000 single-cell ATAC high-quality fragments per cell. Using Signac^63^ and the MACS2 (ref. ^64^) peak caller, we called peaks separately for each cell type, identifying ~119,000, ~104,000 and ~153,000 peaks in PFC L3–L5/L6 IT_*RORB*, L2–L3 IT_*CUX2* and L2–L4 IT_*CUX2*.*RORB* neurons. In the visual cortex, we found ~102,000, 107,000 and 137,000 peaks for the same three cell types (Supplementary Fig. 8a,b). We performed differential peak analysis of matched cell types between the PFC and visual cortex of macaques. Interrogating peaks associated with the set of 3,224 genes targeted for splicing analysis, we found ~2,000 or more differentially regulated peaks for each excitatory subtype (*n* = 1,999, 1,632 and 9,201 for the L3–L5/L6 IT_*RORB*, L2–L3 IT_*CUX2* and L2–L4 IT *_CUX2.RORB* cells, respectively, at an FDR *P* value of 0.05 considering only peaks appearing in at least 2% of cells; Methods and Supplementary Fig. 8c). By contrast, L6 CT/L6b*_SEMA3E*, L5 ET_*GULP1* and L5–L6 NP_*TLL1* neurons showed only two and zero differentially regulated peaks, respectively (Supplementary Fig. 8c). These numbers of differentially regulated peaks between the PFC and visual cortex showed the same trend when displayed as a fraction of significant tests. More specifically, L2–L4 IT_*CUX2.RORB* neurons showed the highest percentage of significant differences (39.71%), far exceeding L3–L5/L6 IT_*RORB* (10.10%) and L2–L3 IT_*CUX2* (9.09%) neurons, with negligible signal in L6_CT/L6b*_SEMA3E* neurons. These results strongly suggested that L2–L4 IT_*CUX2.RORB* neurons have the strongest brain-region-specific chromatin alterations in the vicinity of the enriched set of genes (Supplementary Fig. 8d). Due to varying cell numbers and open chromatin regions, statistical power differed among the excitatory cell types. L2–L4 IT_*CUX2.RORB* neurons had the highest number of cells (4,626 and 9,756 cells in the PFC and visual cortex, respectively) and open chromatin regions. For L3–L5/L6 IT_*RORB* neurons, we observed 2,508 and 3,313 cells in the PFC and visual cortex and 2,153 and 3,776 cells for L2–L3 IT_*CUX2* neurons. To control for statistical power differences, we performed downsampling experiments (Methods) by repeatedly (*n* = 20) sampling 1,000 cells in both regions, calling peaks and choosing the peaks closest to targeted genes and randomly sampling 10,000 peaks among these per region. We performed differential chromatin accessibility experiments as described earlier and recorded the percentage of tests that passed an FDR of 0.05, leading to 20-value distribution of these excitatory neurons. L2–L4 IT_*CUX2.RORB* neurons consistently showed the highest median proportion of significant peaks (~4.9%), which is 2.75× and 3.0× higher than L2–L3 IT_*CUX2* (1.8%) and L3–L5/L6 IT_*RORB* (1.6%) neurons, respectively (corrected two-sided paired Wilcoxon rank-sum test *P* values of <2.87 × 10^−6^ in both cases; Fig. 2i). This result was robust to distinct ways of annotating cells with high-quality chromatin signal (Supplementary Fig. 8e and Methods). To further validate the observation that L2–L4 IT_*CUX2.RORB* neurons are most affected by chromatin alterations with a method that does not depend on statistical testing, we computed the peak similarity for all three cell types in both brain regions using the Jaccard index (Methods). L2–L4 IT_*CUX2.RORB* neurons showed the lowest peak similarity, which again supports its strongest brain region specificity of chromatin regulation (Supplementary Fig. 8f). To assess whether brain-region-specific chromatin changes depend on genomic locations (exonic/intronic/untranslated region (UTR)/intergenic), we performed downsampling experiments (Methods) by randomly sampling 5,000 peaks of each category among all the peaks called from 1,000 cells of each condition per excitatory neuron subtype. Among the three major excitatory neuron subtypes, L2–L4 IT_*CUX2.RORB* neurons showed the highest significance percentage in each peak category, yielding 1.51%, 3.89%, 5.31% and 9.20% as medians for UTR, exonic, intronic and intergenic peaks, respectively (Fig. 2j and Methods). A representative example peak is located in an intron of the gene encoding RNA terminal phosphate cyclase like 1 (*RCL1*), only observed in visual cortex L2–L4 IT_*CUX2.RORB* cells but not in the PFC (Fig. 2k). Notably, the differences observed in open chromatin in specific excitatory subtypes between the two brain regions can lead to PFC-specific occupancy of transcription factors such as *NEUROG1* (Fig. 2l). In summary, chromatin and splicing distinguish matched cell types between the PFC and visual cortex in distinct manners.

### Chromatin cell subtype specificity patterns mimic splicing

**Fig. 3:**
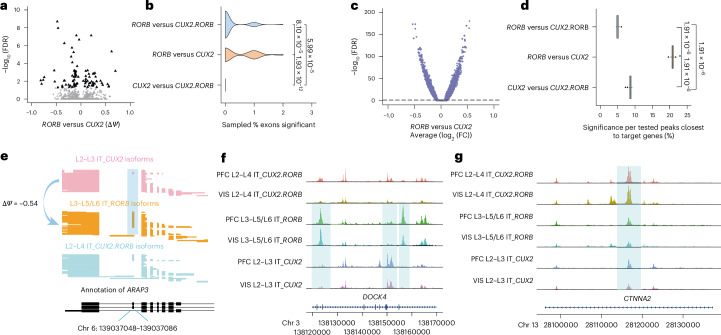
Chromatin accessibility and splicing patterns mimic each other in cell subtypes. **a**, Volcano plot of excitatory neuron subtype-specific splicing comparison of L3–L5/L6 IT_*RORB* versus L2–L3 IT_*CUX2* neurons. **b**, Downsampling experiment. Distribution of the percentage of exons significant in the pairwise subtype comparison in both brain regions (Methods; *n* = 100). **c**, Volcano plot of excitatory neuron subtype-specific comparison of L3–L5/L6 IT_*RORB* and L2–L3 IT_*CUX2* open chromatin regions for three types of excitatory cells; FC, fold change. **d**, Downsampling experiment. Distribution of the percentage of peaks that are significantly different for each pairwise subtype comparison in the vicinity of genes targeted for splicing analysis (Methods; *n* = 20). **e**, Cell-type-resolved single-cell long reads for *ARAP3* plotted. The top three tracks show L2–L3 IT*_CUX2*, L3–L5/L6 IT*_RORB* and L2–L4 IT*_CUX2.RORB* cells, and the bottom black track shows chromosome 6: 139037048–139037086. **f**, Two outer-most peaks that are specific to L3–L5/L6 IT*_RORB* neurons in both the PFC and visual cortex but absent in L2–L3 IT*_CUX2* neurons. The center peak is present in PFC and visual cortex L2–L3 IT*_CUX2* neurons but not in L3–L5/L6 IT*_RORB* neurons. These peaks are in the vicinity of *DOCK4*. **g**, Example peak that is in the vicinity of *CTNNA2* showing increased accessibility only in L2–L4 IT*_CUX2.RORB* neurons in both brain regions. Shading indicates peaks of interest. Each box plot shows the median (middle line), IQR (top and bottom line of the box) and adjacent values (whiskers extending to 1.5× the IQR). Dots represent outliers beyond this range. A two-sided Wilcoxon rank-sum test was applied to all the comparisons shown in **b** and **d**. Adjustments were applied to multiple comparisons, and corrected *P* values (<0.05) are presented.

Because splicing and chromatin profiles can reveal brain region specificities in different ways, we next examined whether they also distinguish excitatory neuron subtypes, regardless of brain region. We performed three pairwise comparisons for differential exon inclusion of L3–L5/L6 IT_*RORB*, L2–L4 IT_*CUX2.RORB* and L2–L3 IT_*CUX2* cells. The L3–L5/L6 IT_*RORB* versus L2–L3 IT_*CUX2* comparison revealed 88 significant exons of 2,705 tested (11 with a | Δ*Ψ* | of ≥0.5; Fig. 3a), whereas the other two comparisons (L2–L3 IT_*CUX2* versus L2–L4 IT_*CUX2.RORB* and L3–L5/L6 IT_*RORB* versus L2–L4 IT_*CUX2.RORB*) showed 0 and 5 significant exons with a | Δ*Ψ* | of ≥0.5 (Supplementary Fig. 9a,b). Downsampling experiments revealed that the L3–L5/L6 IT_*RORB* versus L2–L3 IT_*CUX2* comparison yielded the greatest cell-type differences in exon usage (Fig. 3b; corrected two-sided Wilcoxon rank-sum test *P* values of <8.1 × 10^−5^ ((L3–L5/L6 IT_*RORB* versus L2–L4 IT_*CUX2.RORB*) versus (L3–L5/L6 IT_*RORB* versus L2–L3 IT_ *CUX2*)) and <2.0 × 10^−12^ ((L3–L5/L6 IT_*RORB* versus L2–L3 IT_*CUX2*) versus (L2–L4 IT_*CUX2.RORB* versus L2–L3 IT*_CUX2*))). Cell-type comparisons at the chromatin level (Fig. 3c and Supplementary Fig. 9c,d) also revealed that the L3–L5/L6 IT_*RORB* versus L2–L3 IT_*CUX2* comparison yielded the highest number of differentially accessible peaks using the downsampling strategy (Fig. 3d and Methods; corrected two-sided Wilcoxon rank-sum test *P* < 2.0 × 10^−6^ for all). The total number of exons and percentage of significant exons and peaks mirrored the downsampling trends (Supplementary Fig. 9e–h). Comparing different excitatory neuron subtypes revealed consistent RNA and ATAC patterns, unlike comparisons of the same subtype across brain regions, which showed divergent patterns. As an example of cell-type specificity, an exon of *ARAP3* was included in 61.9% of reads from L3–L5/L6 IT_*RORB* cells but only 7.9% of reads from L2–L3 IT_*CUX2* cells (Fig. 3e). Similarly, *DOCK4* harbors two peaks (chromosome 3: 138122410–138124115 and chromosome 3: 138155707–138157028) exclusive to L3–L5/L6 IT_*RORB* cells and one peak specific to L2–L3 IT_*CUX2* cells across both the PFC and visual cortex (Fig. 3f). However, some peaks also occurred in all subtypes but showed significantly higher accessibility in one subtype, such as in *CTNNA2* (Fig. 3g; chromosome 13: 28116151–28117300). In summary, chromatin and splicing distinguish cell types in a comparable manner when we perform comparisons between neuron subtypes.

### Splicing and chromatin patterns diverge in primate evolution

The rhesus macaque is among the closest common model organisms to humans. Thus, to assess how well macaque chromatin and splicing signatures represent human signatures and whether certain cell types show stronger species-specific divergence in chromatin or splicing is of significance. In addition to the macaque PFC samples used for region comparisons mentioned earlier, we applied ScISOr–ATAC to six human PFC samples (four males and two females) for a species comparison between human and macaque PFC. We sequenced 257 million–427 million Illumina read pairs for the six control RNA libraries (samples C1–C6) and 321 million–367 million for the six ATAC libraries (Supplementary Fig. 10a,d and Supplementary Table 2). RNA profiling revealed multiple cell types and subtypes, with excitatory neurons and oligodendrocytes being the most abundant (Supplementary Fig. 10e,f). Neurons had higher RNA UMI counts, whereas glial cells often had more chromatin molecules (Supplementary Fig. 10g–j). Subtypes within the same cell-type class exhibited UMI abundance variations (Supplementary Fig. 11). Additionally, 42.3 million barcoded, target-gene-enriched long reads were sequenced using ONT for the six human PFC samples. Barcoded reads, UMI counts per cell type and reads reaching a transcription start site or poly(A) site per sample are provided in Supplementary Fig. 12. We integrated the short-read RNA datasets from both species (Methods) and identified 16 cell types and subtypes. Notably, the proportion of L2–L3 IT_*CUX2.CBLN2* cells, L2–L4 IT_*CUX2.RORB.ACAP3* cells and oligodendrocytes differed between human and macaque PFC (Methods, Fig. 1d,e and Supplementary Fig. 13).

**Fig. 4:**
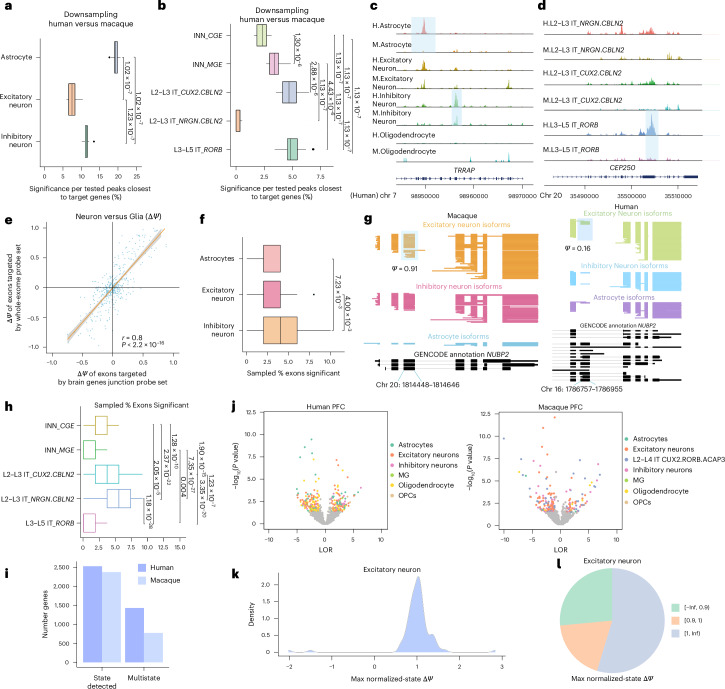
Splicing and chromatin patterns diverge in primate evolution. **a**, Downsampling experiment. Distribution of the percentage of peaks that are significantly different between humans and macaques in the vicinity of genes targeted for splicing analysis per cell type (*n* = 20). **b**, Downsampling experiment for subtypes with the same method described in **a**. **c**, Two peaks within *TRRAP*. The left peak is specific to human astrocytes but is absent in macaque astrocytes, and the right peak shows increased chromatin accessibility in human inhibitory neurons. H, human; M, macaque. **d**, A peak in *CEP250* specific to human L5 IT_*RORB* cells but absent in macaque L5 IT_*RORB* cells. **e**, Correlation between Δ*Ψ* values (neurons versus glia) of tested exons targeted by both exome probes and exon–exon junction probes indicated by regression using a linear model; shading indicates the 95% confidence interval (*n* = 414). Shading indicates peaks of interest. **f**, Downsampling experiment. Distribution of the percentage of exons showing significant differences between humans and macaques per cell type (Methods; *n* = 100). **g**, Cell-type-resolved isoform expression for *NUBP2* plotted, with the top three tracks showing excitatory neurons, inhibitory neurons and astrocytes. **h**, Downsampling experiment for subtypes with the same method described in **f** (*n* = 100). **i**, Number of genes with one or more and two or more cell states detected in both species. Only genes with testable exons were considered. **j**, Volcano plot of state-specific exons across cell types in humans and macaques. Only exons with ten or more reads in two or more states were tested (*n* = 238 and 116 for human and macaque, respectively). Exons with a *P* value of ≤0.05 and | LOR | of >1 are labeled in color, and the others are in gray. A one-sided *χ*^2^ test followed by a Benjamini–Yekutieli multiple testing correction was applied to evaluate the significance of splicing–cell state association. **k**, Distribution of the maximum normalized-state Δ*Ψ* per exon; normalized-state Δ*Ψ* = state Δ*Ψ* /overall Δ*Ψ*. **l**, Pie chart showing the maximum normalized-state Δ*Ψ* per exon split by value into three groups: <0.9, between 0.9 and 1 or ≥1. Each box plot shows the median (middle line), IQR (box) and adjacent values (whiskers extending to 1.5× the IQR). Dots represent outliers. A two-sided Wilcoxon rank-sum test was applied to all the comparisons shown in **a**, **b**, **f** and **h**. FDR correction was applied to multiple comparisons, and corrected *P* values (<0.05) are presented.

We determined corresponding chromatin peaks in macaques and humans and tested these for differential accessibility (Methods). The highest number of significant peaks as a fraction of tested peaks in the vicinity of the 3,224 targeted genes was observed in excitatory neurons, followed by astrocytes and inhibitory neurons (Supplementary Fig. 14a). Downsampling experiments (Methods) showed that astrocytes exhibited the most frequent rearrangements between humans and macaques (Fig. 4a; corrected two-sided Wilcoxon rank-sum test: astrocytes versus excitatory neurons *P* < 1.02 × 10^−7^; astrocytes versus inhibitory neurons *P* < 1.02 × 10^−7^). Highly divergent profiles were observed across neuronal subtypes (Supplementary Fig. 14b). Downsampling experiments revealed the most noticeable species-specific rearrangements in L5 IT_*RORB* excitatory neurons as well as L2–L3 IT_*CUX2.CBLN2* neurons, but much less so in L2–L3 IT_*NRGN.CBLN2* excitatory neurons (corrected two-sided Wilcoxon rank-sum test *P* values of <1.13 × 10^−7^ (L5 IT_*RORB* versus L2–L3 IT_*NRGN.CBLN2*) and <1.13 × 10^−7^ (L5 IT_*CUX2.CBLN2* versus L2–L3 IT_*NRGN.CBLN2*)). In inhibitory neurons, we found a significant difference in peaks between GABAergic interneurons originating from the medial ganglionic eminence (MGE) and the caudal ganglionic eminence (CGE), albeit much less dramatic than between excitatory neuron subtypes (corrected two-sided Wilcoxon rank-sum test *P* value of <1.30 × 10^−6^ (INN_*MGE* versus INN_*CGE*; INN represents inhibitory neuron); Fig. 4b). As an example, a human astrocyte-specific peak and a separate peak specific to inhibitory neurons were identified in *TRRAP* (Fig. 4c). Another example showed a species-specific peak located in one exon of *CEP250* specific to human L5 IT_*RORB* neurons (Fig. 4d). These results indicate that evolution has exerted distinct regulatory effects on chromatin in excitatory neuron subtypes. In our previous work, we demonstrated that genome-wide exome enrichment can successfully remove purely intronic cDNAs from libraries^8,14^. To evaluate the performance of the splice junction-covering enrichment in this study, we compared splice junction versus exome enrichment in two human samples^8^ by calculating Δ*Ψ* values for neurons and glia and found a correlation of 0.8 (*P* < 2.2 × 10^−16^), suggesting high concordance between the two methods (Fig. 4e and Methods).

We previously published methods to assess whether an alignment can be considered consistent with a complete or truncated version of an annotated isoform^65^. Due to more extensive annotation of the human genome than the macaque genome, we found a higher fraction of inconsistent (or novel) long-read RNA alignments in macaques than in humans (Supplementary Fig. 14c). Although fewer significantly differentially included exons were detected in inhibitory neurons than in excitatory neurons (Supplementary Fig. 14d), downsampling revealed the opposite: inhibitory neurons exhibited more frequent species-specific splicing differences than both excitatory neurons and astrocytes (Fig. 4f; corrected two-sided Wilcoxon rank-sum test *P* values of <8 × 10^−3^ (astrocytes versus inhibitory neurons) and 4 × 10^−3^ (excitatory neurons versus inhibitory neurons)). Among neuronal subtypes, splicing showed a trend in opposition to the chromatin analysis. For example, an exon of *NUBP2*, which is conserved between species, was present in 91% of macaque excitatory neuron cDNAs but only in 16% of human excitatory neuron cDNAs (Fig. 4g; two-sided Fisher’s exact test, macaques versus humans, FDR < 6 × 10^−8^).

Among excitatory neuron subtypes, L2–L3 IT_*NRGN.CBLN2* neurons showed the lowest species-specific chromatin arrangements but relatively high species-specific splicing arrangements (Supplementary Fig. 14e). Downsampling experiments confirmed this neuronal subtype to have the highest splicing rearrangements across species among excitatory subtypes (Methods and Fig. 4h; corrected two-sided Wilcoxon rank-sum test *P* values of <2 × 10^−28^ (L2–L3 IT_*NRGN.CBLN2* versus L5 IT_*RORB*) and <2 × 10^−7^ (L2–L3 IT_*NRGN.CBLN2* versus L2–L3 IT_*CUX2.CBLN2*)). Because enrichment probes targeted annotated exon–exon junctions (and given annotation differences between humans and macaques), we tested for potential bias. Importantly, reads often span multiple junctions, reducing bias caused by a single junction missing from the annotation. We compared Δ*Ψ* values between human and macaque excitatory neurons from spliced reads versus those with three or more junctions and found strong correlations (Supplementary Fig. 15a), which remained consistent for reads with four or more, five or more or six or more junctions (Supplementary Fig. 15b–d). In summary, chromatin and splicing analyses show highly divergent results when comparing matched cell types across species. This is especially exemplified by astrocytes that exhibit strong chromatin divergence but limited splicing changes, whereas L2–L3 IT_*NRGN.CBLN2* excitatory neurons show the opposite trend.

To test whether species-specific exon inclusion reflects underlying cell-state differences, we identified cell states for each gene with exons tested in human and macaque PFC comparisons and focused on genes linked to two or more states (Fig. 4i). This revealed many exons whose inclusion was significantly different between cell states in not only the human PFC but also the macaque PFC (Fig. 4j).

Among exons showing species-specific splicing patterns in excitatory neurons, several exons had at least one confirmed observation, defined by a normalized-state Δ*Ψ* of ≥1. The normalized-state Δ*Ψ* distribution appeared larger than in the case of brain regions (Fig. 4k). For 55% of exons in excitatory neurons (64/117), species specificity was confirmed in at least one cell state, whereas 26% had a normalized-state Δ*Ψ* of <0.9, suggesting that these may stem from variations in cell-state abundance (Fig. 4l).

### Chromatin and splicing patterns in AD

To examine whether splicing and chromatin show convergent or divergent cell-type-specific dysregulation in AD, we applied ScISOr–ATAC to ten control PFC samples (six males and four females) and nine AD PFC samples (five males and four females). For the 19 RNA libraries, we sequenced 215 million–479 million Illumina read pairs. For the 19 ATAC libraries, 252 million–512 million read pairs were sequenced (Supplementary Fig. 10). Additionally, we generated >200 million barcoded Agilent target-gene-enriched long reads using ONT technology (Supplementary Fig. 12).

**Fig. 5:**
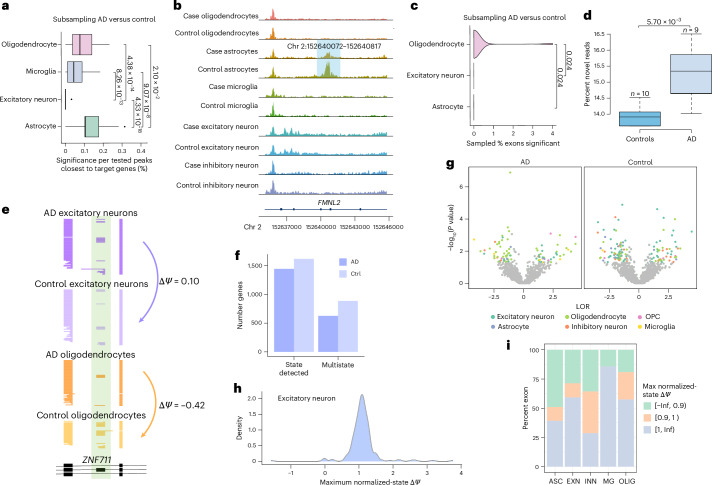
Chromatin and splicing patterns in AD. **a**, Downsampling experiments. The distribution of the percentage of peaks that are significantly different between AD and control samples in the vicinity of genes targeted for splicing analysis is shown (Methods; *n* = 20). **b**, A peak that is highlighted within *FMNL2* that is present in control astrocytes but not in AD astrocytes. Shading indicates peaks of interest. **c**, Downsampling experiment. The distribution of the percentage of exons showing significant differential inclusion per cell type in AD versus control is shown (Methods; *n* = 100). **d**, Percentage of novel reads found within control (*n* = 10) and AD (*n* = 9) datasets. **e**, Cell-type-resolved single-cell long reads for *ZNF711*. The top two tracks show AD excitatory neurons and control excitatory neurons, followed by AD oligodendrocytes and control oligodendrocytes. The bottom black track shows chromosome X: 85264898–85268508. **f**, Number of genes with one or more and two or more cell states detected in AD and control samples. Only the genes with testable exons were considered for cell-state detection. **g**, Volcano plot of state-specific exons across multiple cell types in AD and control groups (only exons with ten or more reads in two or more cell states were tested; *n* = 494 and 726). Exons with a *P* value of ≤0.05 and | LOR | of >1 are labeled in color, whereas the others are labeled in gray. A one-sided *χ*^2^ test followed by Benjamini–Yekutieli multiple testing correction was applied to evaluate the significance of splicing–cell state association. **h**, Density plot of the distribution of the maximum normalized-state Δ*Ψ* per exon. Normalized-state Δ*Ψ* = state Δ*Ψ* /overall Δ*Ψ*. **i**, Stacked bar plot showing the proportion of maximum normalized-state Δ*Ψ* per exon split by value into three groups: <0.9, between 0.9 and 1 or ≥1. The ‘≥1’ group represents the fraction of disease-associated overall Δ*Ψ* values, which can be seen in specific cell states by cell type. Each box plot shows the median (middle line), IQR (box) and adjacent values (whiskers extending to 1.5× the IQR). Dots represent outliers. A two-sided Wilcoxon rank-sum test was applied to all comparisons shown in **a**, **c** and **d**. FDR correction was applied to multiple comparisons, and corrected *P* values (<0.05) are shown.

We found that oligodendrocytes, and to a lesser extent astrocytes, exhibit numerous chromatin changes in AD. In total, 1,480 peaks (22.13%) near splicing-targeted genes showed significant changes in oligodendrocytes, whereas neurons showed «1% of such changes, possibly due to survival bias (Supplementary Fig. 16a,b). Furthermore, downsampling experiments (Methods) revealed a clear trend in which astrocytes were most affected in AD, followed by oligodendrocytes and microglia, whereas excitatory neurons showed the lowest effects (Fig. 5a; corrected two-sided Wilcoxon rank-sum test *P* values of <4.4 × 10^−14^ (oligodendrocytes versus excitatory neurons), <8.3 × 10^−13^ (microglia versus excitatory neurons) and <4.4 × 10^−18^ (astrocytes versus excitatory neurons)). For example, a peak located next to two exons in *FMNL2* was specifically lost in astrocytes in AD (Fig. 5b). On the splicing side, excitatory neurons showed the highest fraction of dysregulated exons (Supplementary Fig. 16c,d and Supplementary Table 6). However, downsampling experiments (Methods) revealed that oligodendrocytes showed the strongest dysregulation, whereas the other cell types did not (Fig. 5c; two-sided Wilcoxon rank-sum test *P* values of <3 × 10^−2^ (oligodendrocytes versus excitatory neurons) and <3 × 10^−2^ (oligodendrocytes versus astrocytes)). To validate this downsampling procedure for both chromatin and splicing, we used positive and negative controls. In a positive control (neurons versus glia), downsampling correctly identified significant changes. In a negative control (neurons split randomly), no differences were detected, confirming the method’s specificity (Supplementary Fig. 17a,b). Furthermore, SynGO analysis of splicing differences between neurons and glia revealed nearly identical top categories in both the full and downsampled datasets (Supplementary Fig. 17c), supporting the method’s reliability. We then probed AD and control reads for how often they were inconsistent or truncated with any annotated isoform^65^. Notably, AD samples showed a higher inconsistency (or novelty) fraction (Fig. 5d), a difference not explained by intron number per read (Supplementary Fig. 18).

In AD versus controls, we observed an oligodendrocyte-specific dysregulated exon of the gene encoding zinc finger protein 711 (*ZNF711*), a transcriptional regulator of neuron development that is associated with X-linked intellectual disability^66,67^. Oligodendrocytes showed a 42% decrease in exon inclusion in AD, whereas excitatory neurons showed a 10% increase in AD (Fig. 5e). To support dysregulation of splicing in AD oligodendrocytes, Gene Ontology analysis of dysregulated genes at the chromatin level revealed multiple splicing- and RNA biology-linked terms (Supplementary Fig. 19a). In neurons, splicing alterations in AD were functionally distinct: excitatory neurons were more linked to postsynaptic roles, whereas inhibitory neurons were presynapse-term dominated (Supplementary Fig. 19b). Significantly dysregulated exons did not stand out in terms of exon length, being entirely coding or maintaining the reading frame (Supplementary Fig. 19c–e). On the chromatin side, peaks dysregulated in oligodendrocytes in AD samples were mostly found in UTRs and introns (Supplementary Fig. 19f,g). In summary, both splicing and chromatin are most strongly altered in AD in glia, especially oligodendrocytes.

Because previous results showed that cell state influences splicing, we asked if splicing differences in AD could be driven by such states. Among the genes tested for such differences in splicing, we identified one or more cell states per gene for approximately 1,500 genes in both conditions (Fig. 5f). Many exons showed inclusion differences associated with cell states in both AD and control samples (Fig. 5g). Among excitatory neurons, and focusing on exons with detected AD-specific inclusion, we normalized the maximum state-specific **Δ***Ψ* by the overall **Δ***Ψ* and observed a symmetric distribution centered around 1. Thus, confirming states could be found for many exons, but not for all (Fig. 5h, Supplementary Fig. 20 and Methods). We performed this analysis for other cell types and found that astrocytes and oligodendrocytes stood out as having relatively low and high ratios of exons with maximum normalized-state-specific **Δ***Ψ* values of ≥0.9, respectively (Fig. 5i).

## Discussion

Measurements of multiple modalities have become commonplace in single-cell genomics. Here, we introduce ScISOr–ATAC, which enables the simultaneous recording of splicing patterns and open chromatin states in frozen samples.

From a systems biology perspective, multimodal measurements help determine whether one or more modalities influence a third and whether different modalities provide converging or diverging views of cell-type diversity. In comparing macaque brain regions, we found that distinct cell chromatin–transcriptome states can result in different exon inclusion outcomes. This raises questions about how these influences are mediated between cell state and splicing and whether these may underlie splicing differences. ScISOr–ATAC allows us to assess the extent of these effects. We examine this across three contexts: matched cell types in macaque PFC and visual cortex, human–macaque PFC divergence and cell-type-specific dysregulation in AD.

Although the PFC and visual cortex are both cortical regions that engage separate functions, both regions do harbor transcriptionally similar excitatory neuron subtypes. Here, we distinguish three such excitatory neuron subtypes: *RORB*^+^*CUX2*^−^, *CUX2*^+^*RORB*^−^ and *RORB*^+^*CUX2*^+^. Brain-region-specific splicing is most evident in L3–L5/L6 IT_*RORB* neurons, whereas chromatin differences are more pronounced in L2–L4 IT_*CUX2.RORB* neurons. This highlights how each modality captures unique aspects of regional identity, reinforcing the value of multimodal approaches. Of note, some brain-region-specific exon inclusion events co-occur with cell state arrangements. However, most brain-region-specific splicing events can be validated by one or more cell states.

Within the same brain region, ATAC and splicing patterns often highlight similar cell subtype distinctions, although they sometimes reveal unique features. Comparing human and macaque PFC, astrocytes show major chromatin differences but conserved splicing, whereas L2–L3 IT_*NRGN.CBLN2* neurons display the opposite. In terms of biological evolution, the above finding shows that distinct cell types have undergone evolutionary changes in different modalities. Similar to the brain region comparison, species-specific splicing patterns are often identified within one or more shared cell states, supporting their validity. However, we also observe many examples where the overall observation of species-specific splicing cannot be seen in any cell state, which could be caused by underlying cell-state differences per species.

In the case of AD, astrocytes show strong AD-related dysregulation in chromatin, but not in splicing, highlighting modality-specific effects. Many AD-associated splicing changes are reproducible across cell states, suggesting true dysregulation rather than cell-state differences. The weaker neuronal signals may stem from survival bias, where severely affected neurons are under-represented due to cell loss. Additionally, our results indicate the cell types that undergo splicing dysregulation. Although often dysregulation correlates between pairs of cell types, examples are cell-type specific. The cell-type-specific dysregulated splicing events detected in our AD study can serve as underlying therapeutic targets in the future.

We also observed that chromatin and splicing show divergent patterns in the species comparison and convergent patterns in the AD pathology analysis, possibly due to different timescales. In AD, molecular changes normally could unfold in several years, allowing persistent interactions across modalities (for example, chromatin opening might upregulate splicing factors). By contrast, evolutionary divergence over millions of years may lead to genomic rewiring that decouples these relationships. Additionally, although chromatin and splicing are linked through co-transcriptional processes^61,62,68–75^, it is still a challenge to perfectly predict the impact of chromatin changes on splicing.

Additionally, our current work analyzes chromatin and RNA from the nucleus. Nuclear RNA has advantages and disadvantages, which we discussed recently^76^. In brief, nuclear RNA is less likely to yield full-length isoforms due to internal oligo(dT) priming. On the upside, nuclear RNAs allow for the detection of incomplete spliced cDNAs derived from very long mRNAs that were undergoing RNA processing. In cytosolic preparations, some such genes might be biased against because the resulting full-length cDNA is simply too long for amplification and sequencing.

ScISOr–ATAC faces limitations primarily due to the challenges of long-read sequencing, including high cost and lower depth than short-read data. Limited depth can undermine performance, especially for downsampling analyses, which require a minimum read number per exon per cell type. Deeper sequencing enables more exons and cell types to be considered. Moreover, because splicing can vary by cell state, future studies aiming to track splicing across cell-state transitions would benefit from higher depth and more affordable long-read sequencing.

In summary, these findings highlight the advantages of simultaneous measurements of chromatin and splicing in state-of-the-art neuroscience approaches as they often show divergent patterns. Additionally, we demonstrate that splicing can be influenced by cell state, re-enforcing the need for multimodal datasets. Furthermore, we provide a detailed map of cell-type specificity of chromatin and splicing across brain regions, species and disease.

## Methods

### Ethics statement

All experiments were conducted in accordance with the 2011 Eighth Edition of the NIH Guide for the Care and Use of Laboratory Animals. Animal procedures were performed according to protocols approved by the Animal Care and Use Committee of Rockefeller University.

### Macaque brain tissue acquisition

Brains were collected from two adult male rhesus macaques (M1 and M2, ages 29 and 26) that were humanely killed via intramuscular administration of ketamine, followed by intravenous administration of a pentobarbital overdose for approximately 10 min. These primates had not been exposed to any experimental pharmacological treatment for ≥6 months before being killed and had no recorded infections. Brains were collected within 20 min after pentobarbital administration (post mortem interval: 2 h and 1 h), placed on ice and dissected into 5- to 10-mm coronal slices of PFC and visual cortex using a brain mold guided by the Allen Brain Atlas. Samples were flash-frozen and maintained at −80 °C until processing.

### Human brain tissue acquisition

All human samples were deidentified postmortem frozen samples, which were requested from the tissue banks maintained by the Center for Neurodegenerative Disease Research (CNDR) and the University of Pennsylvania Alzheimer’s Disease Core Center (ADCC), according to Weill Cornell Medicine institutional review board-approved protocols. Sample collection was conducted by CNDR/ADCC. A total of nine PFC samples from individuals with AD (five males and four females) and ten control PFC samples from individuals not diagnosed with dementia (six males and four females) were included in this study. Participant sex, age and diagnosis information was supplied by CNDR/ADCC and can be found in Supplementary Table 2. This study is considered ‘non-human subject research’.

### Single-nuclei isolation

Single-nuclei isolation was performed for fresh-frozen human brain samples using the SnISOr–Seq^8^ protocol and the ATAC–seq protocol published by Corces et al.^77^.

### 10x Single-nuclei cDNA generation, gene expression and ATAC library construction and Illumina sequencing

A 10x Multiome ATAC + Gene Expression assay was performed by following the manufacturer’s instructions (10x Genomics, CG000338_ChromiumNextGEM_Multiome_ATAC_GEX_User_Guide_RevE, Chromium Next GEM Single Cell Multiome Reagent Kit A, 16 reactions PN-1000282). The quality of full-length 10x cDNA, ATAC and 3′ gene expression short-read libraries was measured by Qubit dsDNA HS assay (Invitrogen, Q32854) and TapeStation Genomic DNA assay (Agilent, 5067-5365 and 5067-5366). Sequencing libraries were loaded on Illumina NovaSeq6000 with PE 2 × 100 paired-end kits by setting the following read length: 28 cycles read 1, 8 cycles i7 index and 91 cycles read 2 for gene expression libraries and 50 cycles read 1N, 8 cycles i7 index, 24 cycles i5 index and 49 cycles read 2N for ATAC libraries. The fastq files were generated by running bcl2fastq v2.20.

### Linear/asymmetric PCR and exome capture

Linear/asymmetric PCR was applied to naive full-length 10x cDNA derived from the last step to remove the nonbarcoded cDNA. Spliced barcoded cDNA was enriched by performing exome capture using custom SureSelect probe sets designed for macaques/humans and the reagent kit SureSelectXT HSQ (Agilent, G9611A). The detailed linear/asymmetric PCR and exome capture protocol is described in the SnISOr–Seq pipeline^8,14^.

### Library preparation for ONT

For all samples, ~75 fmol of cDNA processed with linear/asymmetric PCR and exome capture underwent ONT library construction by using a Ligation Sequencing kit (Oxford Nanopore, SQK-LSK110 and SQK-LSK114) according to the manufacturer’s protocol (Nanopore Protocol, Amplicons by Ligation). The ONT library was loaded onto a PromethION sequencer by using a PromethION flow cell (Oxford Nanopore, FLO-PRO002 and FLO-PRO114M) and sequenced for 72 h. ONT long reads were base called using MinKNOW 20.06 or MinKNOW 23.07 and filtered for a base quality score of >7.

### Exon–exon junction probe design

A list of 3,630 human genes (3,224 ortholog genes in macaques), including synaptic genes^46^ (659 for macaques and 720 for humans), TDP-43 binding targets^47^ (30 for macaques and 33 for humans), genes with cell-type-specific highly variable exons in the human PFC^8^ (259 for macaques and 271 for humans) and genes associated with missplicing in AD^16^ (173 for macaques and 202 for humans), ASD^48–50^ (1,875 for macaques and 2,102 for humans), ALS^52^ (391 for macaques and 428 for humans) and schizophrenia^51^ (962 for macaques and 1,080 for humans), was assembled. Using the GENCODE human annotation (release 34)^78^, all protein-coding transcripts of these genes were identified. For each exon–exon junction present in at least one transcript, 140 bases spanning the junction were selected, with 70 exonic bases on either side. If an exon was shorter than 70 bases, adjacent exon sequence was included to reach the required length. Sequences shorter than 130 bp or mapping to more than five genomic loci were excluded. Genes with fewer than five valid probes were also removed. A 120-mer was chosen from within the initial (130- to 140-base) sequence using Agilent Technology’s method for maximizing hybridization efficiency.

### Short-read data processing

Both RNA and ATAC fastq files of the M1 PFC sample were subsampled randomly using seqtk 1.3 (https://github.com/lh3/seqtk) to reach a close reads per cell number with the other three samples. The cellranger-arc reference for macaques was built based on the gene annotation of mulatta.Mmul_10 release 104 and genome assembly of Mmul_10 downloaded from Ensembl^79^. The cellranger-arc reference for human was downloaded from 10x Genomics (References-2020-A Human reference, GRCh38).

### Gene expression data processing and cell-type annotation

Gene × cell matrices processed with cellranger-arc-2.0.1 (refs. ^80,81^) were loaded into Seurat 4.2.0 (refs. ^37^), and cells were filtered per sample. Doublets were removed before clustering with DoubletFinder 2.0.3 (ref. ^39^) with an expected doublet ratio of 8~16%. After filtering for high-quality cells, each sample was scaled and normalized using default parameters and clustered using Seurat^37^. All samples from the same species were merged, scaled and normalized, and variable features were identified. Batch effect correction was performed using Harmony^38^. Cells were annotated based on published cell-type markers^31,32^, as well as the Azimuth^37^ human dataset and other published datasets (https://compbio.mit.edu/ad_aging_brain/) as references^82,83^. The marker genes used for cell-type/subtype annotation are shown in Supplementary Figs. 1 and 10.

### Differential gene expression analysis

For each cell type/subtype, the set of differentially expressed genes detected from the comparison between conditions (macaque visual cortex versus PFC) was obtained by running the FindMarkers function of Seurat^37^ (test = MAST, FDR < 0.05, | log_2_ (fold change) | > 0). Gene Ontology enrichment analysis for the differentially expressed genes was performed by using the enrichGO function of clusterProfiler^84^ 4.2.2 (OrgDb = org.Mmu.eg.db, pAdjustMethod = ‘BH’).

### Compositional data analysis for cell types

Compositional data analysis for cell types identified in the species comparison between human and macaque PFC samples was performed using scCODA 0.1.9 (ref. ^85^), and results are shown in Supplementary Fig. 14d.

### ATAC data processing

Fragments and peak × cell matrices processed with cellranger-arc-2.0.1 were loaded into Signac^63^ and Seurat^37^, and each sample was preprocessed individually with a unified set of peaks generated from bed files of all four samples to build the ‘ATAC’ assay. High-quality cells were selected after quality control and doublets removal using Signac^63^ and DoubletFinder 2.0.3 (ref. ^39^). Subsequently, normalization and dimensional reduction were performed after sample merging and batch effect correction using Harmony^38^. We used two methods to perform cell-type annotation for ATAC data. (1) Cells with matched barcodes in both single-cell RNA-seq and single-cell ATAC–seq data were retained using the barcode translation output from cellranger-arc-2.0.1, and cell-type identities from single-cell RNA-seq were directly assigned to corresponding single-cell ATAC–seq cells. This method was applied to all peak-related figures except for S8e. (2) Single-cell ATAC–seq cells were annotated via label transfer using Signac^63^. This method was applied to S8e only. The peaks were called per cell type/subtype using MACS2 (ref. ^64^) by running the CallPeaks function of Signac^63^. The ‘Peak’ assay was built for downstream analysis using the Signac functions FeatureMatrix and CreateChromatinAssay. The annotation object supplied for CreateChromatinAssay^63^ was built based on the gene annotation of mulatta.Mmul_10.104 (macaque) or Hsapiens.v86.annotation.hg38 (human) released by Ensembldb. Peaks found in >2% of cells and located on standard chromosomes were tested for differential accessibility between conditions (test method = LR, log (fold change) cutoff = 0), among which the peaks with an FDR of <0.05 were considered significant. Using the Grange files generated by reading the Ensembl-based annotation of macaques/humans with the function import.gff (rtracklayer V1.54.0)^86^, peak annotation was performed by running the ClosestFeature function of Signac_1.2.1 (ref. ^63^) or bedtools closest (V2.30.0)^87^ with the bed-formatted gene annotation transformed by the gtf2bed function of BEDOPS V2.4.41 (ref. ^88^). The ratios of significant peaks closest to the target genes were calculated as the peaks with an FDR of <0.05 among the peaks closest to the genes targeted in the splicing analysis.

### Evaluate differential accessibility between conditions/cell types by downsampling

Peak calling, normalization, batch effect correction, differential accessibility analysis and generation of the peak annotation pipeline were performed as described in ‘ATAC data processing’ for all downsampling experiments. All the related box plots, scatter plots and density plots were generated using ggplot2 (ref. ^89^).

### Brain region comparison and species comparison

For each cell type or subtype, 1,000 cells from each condition were randomly sampled. For condition comparisons (macaque PFC versus visual cortex or human PFC versus macaque PFC), 10,000 peaks were randomly subsampled among all peaks called from 2,000 cells (sum of cells from both conditions) per cell type or subtype and differential accessibility of peaks that were found in >2% of cells were tested (test method = LR, log (fold change) cutoff = 0, FDR < 0.05). Subsampling was repeated 20 times.

### Excitatory neuron subtype comparison

For each pair of excitatory neuron subtypes shown in Fig. 3d, 10,000 peaks were randomly subsampled from the peaks called from 4,000 cells (1,000 cells of each subtype per brain region) per subtype comparison, and the differential accessibility of peaks observed in >2% of cells was tested (test method = LR, log_2_ (fold change) cutoff = 0, FDR < 0.05). Random subsampling was repeated 20 times.

### Human AD versus control

To evaluate the differential accessibility per major cell type between AD and control samples, we randomly chose seven of ten control samples and six of nine AD samples for downsampling. For 7 random control samples, 150 random cells were selected per sample to make a total of 1,050 cells as the control group. Similarly, for 6 random control samples, 175 random cells were selected per sample to make a total of 1,050 cells as the AD group. For the condition comparison between AD and control samples, peaks were called from 2,100 random subsampled cells per cell type, followed by random sampling of 20,000 peaks for the differential accessibility test. Only peaks that were detected in >2% cells were tested (test method = LR, log (fold change) cutoff = 0, FDR < 0.05). Subsampling was repeated 20 times.

### Neurons versus glia and comparison within neurons (human control PFC)

This experiment was performed as a proof of concept for downsampling shown in Supplementary Fig. 17b. The same protocol performed for AD versus control samples was applied to evaluate differential accessibility in neurons versus glia (positive control) and within neurons (negative control). Subsampling was repeated 100 times.

### Differential accessibility of different peak categories between conditions evaluated by downsampling

To evaluate chromatin accessibility differences between excitatory neuron subtypes by genomic location, peaks were divided into different categories (exon/intron/UTR/intergenic) according to the closest features defined by the annotation. Of note, only the peaks whose closest features were either protein-coding genes or long noncoding RNA genes and that were located on standard chromosomes were considered here. For each peak category, 5,000 peaks were randomly selected from the peaks called from 1,000 cells randomly subsampled per condition (PFC/visual cortex). Random subsampling was performed 20 times.

### Evaluation of the similarity of cell-type-specific peak sets of different conditions with the Jaccard similarity index

For each excitatory neuron subtype (*RORB*, *CUX2* and *CUX2.RORB*), peaks were called for PFC or visual cortex cells separately. The peak calling, normalization and batch effect correction pipeline was performed as described in ‘ATAC data processing’. The peak coordinates were exported using granges function of GenomicRanges 1.46.1 (ref. ^90^) and written in sorted bed format. The Jaccard similarity index of the comparison between peaks called from PFC and visual cortex cells of each excitatory cell type was calculated using the bedtoolsr::bt.jaccard function of BedtoolsR 2.30.0-5 (ref. ^91^).

### Differential motif enrichment analysis

For each excitatory neuron subtype (*RORB*, *CUX2* and *CUX2.RORB*), we used the getMatrixSet and AddMotifs functions of Signac^63^ to get the motif information. Overrepresented motifs (FDR < 0.05) were detected by setting the significant brain-region-specific peaks as background (parameters for finding differentially accessible peaks: FDR < 0.05, test method = LR, min.pct = 0. 02, | log_2_ (fold change) | > 0). The enrichment score violin plot of one of the top hits is shown in Fig. 3i.

### Mapping orthologous exons in human data

The TransMap^92^ projection alignment algorithm was used to map exons between human and macaque assemblies. LASTZ^93^ 1.04.15 genomic alignments between the human GRCh38 and macaque RheMac10 reference assemblies were used to map reference transcript annotations between assemblies. TransMap was used instead of UCSC Genome Browser liftOver^94^, as it produces base-level alignments, allowing observation of indels and other differences between the LASTZ chain and net alignments files. These were obtained from the UCSC Genome Browser site, along with the below-mentioned programs to process them. Syntenic genomic alignments were obtained by filtering the net files to obtain the syntenic nets using ‘netFilter -syn’ and then using ‘netChainSubset -wholeChains’ to obtain a set of syntenic chain alignments for mappings. GENCODE^78^ human v35 and macaque were mapped to the other assembly using the ‘pslMap’ program^95^.

### Species comparison of peaks in conserved exons

The most conserved exons pairs between humans and macaques were considered for chromatin accessibility comparison. A total of 157,596 human exons and 157,562 macaque exons composed of 159,279 pairs, which indicates for each exon in one species, only the one with the highest ortholog similarity in another species was considered. For all normal human PFC samples, the command bedtools intersect^87^ was used to filter for the fragments that overlapped with human exons (≥1 bp) in the conserved exon pairs. The conserved exon-covering fragments were sorted and indexed for each sample. The same procedure was performed for all macaque PFC samples except for that fragments were mapped to the hg38 genome by rtracklayer::liftOver^96^. For the bed file of ATAC peaks in Cell Ranger output, only the peaks covering conserved exons were kept for each sample. Similar to the fragment file processing procedure, all macaque PFC sample peaks were mapped to the human hg38 genome and combined with human PFC sample peaks. The conservative exon-covering peaks and fragments were used for ATAC assay creation. To build the ‘Peak’ assay, the peaks were called either by major cell types or subtypes by MACS2 (ref. ^64^; by running the CallPeaks function of Signac^63^). By running the Signac functions FeatureMatrix and CreateChromatinAssay, the ‘Peak’ assay was built for downstream analysis. The annotation used for CreateChromatinAssay^63^ was built based on the human gene annotation EnsDb.Hsapiens.v86. Only standard chromosome peaks were considered. Similarly, the combined data of the ‘Peak’ assay were scaled and normalized, and the top features were identified. Integration of data to control for sample-specific batch effects was performed using Harmony^38^.

### Long-read data processing

ONT fastq files were first filtered for barcoded reads with the GetBarcodes function from scisorseqR^9^. Reads were mapped using minimap2 (ref. ^53^), followed by differential splicing analysis with scisorseqR using the commands MapAndFilter() and InfoPerLongRead() with default settings. Given that the default setting of the command InfoPerLongRead() requires a ‘minTimesIsoObserve’ equal to 5, only the spliced reads that support the unique isoforms observed at least five times were kept and recorded in AllInfo files of each sample. The generated AllInfo files were then UMI corrected, where UMIs were required to have an edit distance of ≥4. If multiple reads with similar UMIs did not meet this criterion, then only one read of the group was kept. UMI-filtered AllInfo files were used in scisorATAC’s casesVcontrols function with basic settings to yield differentially spliced exons. The cell-type-resolved single-cell long-read assignments per example gene with alternative exons were plotted using ScISOrWiz^59^.

### Validation of *POLN* exon inclusion using quantitative PCR with reverse transcription

RNA was extracted from macaque tissue isolated from the PFC or visual cortex using an RNeasy Mini kit (Qiagen, 74104), which involved on-column DNase I digestion before RNA elution. cDNA was synthesized using SuperScript IV Reverse Transcriptase (Invitrogen, 18090200), according to the manufacturer’s protocol. Quantitative PCR with reverse transcription was performed using 30 ng of cDNA as template per sample, validated primers (see below) and PowerUp SYBR Green Master Mix (Applied Biosystems, A25742) on a QuantStudio 3 Real-Time PCR System (Thermo Fisher Scientific). Primers for quantitative PCR with reverse transcription were designed by using Primer-BLAST and were synthesized by Thermo Fisher Scientific. The primers targeted mutually shared *POLN* exons (5′-TGAGCAGTAACCAGCTTCGAG-3′ and 5′-GATGAAGGTCTCGCAGAGCA-3′) or visual cortex-specific exons (5′-AGAGTAGAGTCAGGGAGCCA-3′ and 5′-TGCCTCCTGGGTTCAAGCGA-3′). Comparisons were made using the comparative cycling threshold (*C*_t_) method, and data were normalized to the PFC and are shown as fold change.

### Merge macaque and human expression data by liger

We used liger^97^ to integrate the RNA assay data from six human normal PFC samples and two macaque PFC samples. We annotated a total of 16 cell types using the pipeline described in the gene expression data analysis section. Among all nine excitatory neuron subtypes, we only considered the three most abundant subtypes, which were L2–L3 IT_*CUX2.CBLN2*, L2–L3 IT_*NRGN.CBLN2* and L3–L5 IT_*RORB* cells, for species comparison. The other two abundant subtypes (L2–L4 IT_*CUX2.RORB* and L2–L4 IT_*CUX2.RORB.ACAP3* (2,843 and 4,549)) were excluded from the following analysis as they are under-represented in the human samples. Additional excitatory neuron subtypes (L5/L6 NP, L5 ET, L6 CT/L6b and L6 IT CAR3/L6 IT) were recovered in both species but were also excluded as each comprise less than 1,000 cells.

### Calling differentially included exons

For each cell type and alternative exon, inclusion counts and exclusion counts were collected as previously performed. Before testing for differential exon inclusion, a *χ*^2^ criterion was applied for filtering. To compare exon inclusion for two distinct comparisons, a 2 × 2 table was populated for inclusion and exclusion counts for the two conditions, and a two-sided Fisher’s exact test was used following a Benjamini–Yekutieli correction for multiple testing. See the Supplementary tables for lists of significant excitatory subtype exons between the PFC and visual cortex.

### Downsampling experiments for differential splicing analysis

#### Downsampling splicing experiments for the PFC versus visual cortex, PFC cell-type comparison and species comparison

To compare two comparisons (that is, differences between the PFC and visual cortex in *RORB*^+^ cells against the same areas in *CUX2*^+^ cells) with equal power, we performed downsampling experiments. We selected all exons that had at least 20 exclusion or inclusion counts in both brain areas. This was followed by randomly selecting 20 reads among the total. These reads were then used to recalculate the difference in percent isoform inclusion between the areas (**Δ***Ψ*). Next, we selected 100 exons randomly for this cell type between two brain areas, enforcing that there be at most one exon per gene. We then repeated these steps for all cell types that were compared. This yielded 100 2 × 2 tables for all comparisons, with exactly equal column sums and the same characteristics (table number) for multiple testing correction. We then performed a Fisher’s exact test and Benjamini–Yekutieli correction for multiple testing and recorded the number of significant events for all comparisons. The procedure was repeated 100 times, giving a distribution of significant percentages for both comparisons. These two distributions were compared with a two-sided Wilcoxon rank-sum test. For disease downsampling in Fig. 2f, we used 20 exons rather than 100 due to smaller sample size. This process was done for all downsampling comparisons except for AD versus control samples in Fig. 5 to account for individual variation due to the high number of individuals that were used. We describe this process below.

#### Downsampling splicing experiments for AD versus control samples

To account for individual variation due to the high number of human samples involved in this analysis, we designed an updated downsampling process to equalize comparisons and ensure that observed changes were not contributed by one or a few individuals. In this process, we first selected exons that were observed in seven or more samples in both the AD and control groups. We then randomly selected seven of those available samples to use in this analysis. We next filtered all exons that had five or more reads per sample, yielding a minimum of 35 reads per group. We then randomly selected 4 reads per sample, yielding a total of 28 reads per group. Twenty-five exons per cell type with sufficient data were selected randomly between AD and control samples. This yielded 25 2 × 2 tables for all comparisons, with exactly equal column sums and the same characteristics (table number) for multiple testing correction. We then performed a Fisher’s exact test and Benjamini–Yekutieli correction for multiple testing and recorded the number of significant events for all comparisons. The procedure was repeated 100 times, giving a distribution of significant percentages for cell types. These distributions were compared with a Wilcoxon rank-sum test.

#### Downsampling splicing experiments for neurons versus glia and comparisons within neurons

This experiment was performed as a proof of concept in control human PFC data and is shown in Supplementary Fig. 17a. For the neuronal control group (neuron 1 versus neuron 2), all neuronal cell types were combined and split into two equal groups (neuron 1 and neuron 2). In comparison, we compared all neurons to all glia. Downsampling experiments were performed the same as in the PFC versus visual cortex, PFC cell-type comparison and species comparison sections.

### Comparing SynGO terms shared by neurons versus glia splicing analysis and downsampled analysis

To validate the downsampling analysis shown in Supplementary Fig. 17c, we compared the Gene Ontology of differentially spliced genes from both the full splicing and downsampling analyses.

### Exome capture efficiency comparison between probe sets

To compare the exome capture efficiency between different probe sets, we used two datasets derived from two human PFC samples (C4 and C6), which were exome captured by two probe sets (whole-exome probe and brain gene exon–exon junction probe) separately. The whole-exome probe-captured dataset was released in our previous publication^8^. For each dataset, the differential splicing analysis was performed by comparing neurons and non-neurons by running the ‘casesVcontrols’ function of the scisorATAC package. The correlation between the Δ*Ψ* values of shared tested exons derived from two datasets is shown in Fig. 4e.

### Statistical sensitivity simulations

We made large numbers of matrices with a Δ*Ψ* of 0.1 (1,000 total counts in each column). All such matrices have *P* values of ≤10 × 10^−^. We then downsampled these to combined counts of 0–9 (in each column), 10–19 (in each column), 20–29 (in each column), 30–39 (in each column), 40–49 (in each column) and 50–249 (in each column) and recorded the fraction of matrices that passed Benjamini–Yekutieli correction for multiple testing (at a corrected *P* value of <0.05 for 100 tests in each case). These fractions give an idea of how many reads are required to find a true Δ*Ψ* of 0.1. We repeated this process for Δ*Ψ* 0.2, 0.3, 0.4 and 0.5. In summary, for a Δ*Ψ* of ≥0.4 and read numbers (in each column) of ≥30, one reaches a sensitivity of 82%. These data are shown in Supplementary Fig. 6e.

### Correlation between cell-type-specific splicing and cell states revealed by transcription and chromatin accessibility

To get the cell states defined by transcription and chromatin accessibility per gene per cell type, we followed the tutorial of Velocyto^98^ and MultiVelo^10^ 0.1.3. Loom files were obtained by running Velocyto 0.17 for all human PFC samples (ten control and nine AD samples) and macaque samples (two PFC and 2 visual cortex). With the spliced and unspliced counts stored in loom files, running MultiVelo velocity stream and latent time was performed for the genes that had exons tested for differential splicing of each comparison (1,571 genes for the macaque brain region comparison, 2,936 genes for the species comparison and 1,874 genes for the AD versus control comparison).

A state value of 0, 1, 2 or 3 (corresponding to cell states priming, coupled-on, decoupled and coupled-off, respectively) was assigned to each cell × gene pair by Multivelo based on the RNA-seq and ATAC–seq expression dynamics. These state values (*S*_gc_ ∈ {0,1,2,3}) were then used to connect to the exon splicing levels per cell. If a gene only exhibited one state across all cells, then it was classified as a single-state gene and excluded from further analysis. For each cell in a given cell type, and all tested exons for a particular condition, we used the UMI-corrected AllInfo files as input to obtain the inclusion or exclusion of an exon–cell pair. Using the state value assigned for a gene (*S*_gc_) as a proxy for all exons in that gene (*S*_ec_), the exon inclusion and exclusion vectors for a cell type were decomposed into individual state vectors. Thus, a matrix containing the state values as rows and inclusion or exclusion values as columns was populated. A state-wise percent spliced in (*Ψ*) value was therefore obtained by dividing the inclusion counts for a state by the total number of molecules arising from that gene containing that state. A matrix was only considered for testing for differential inclusion if it fulfilled the *χ*^2^ criteria. A *P* value using the *χ*^2^ test was reported, and if the number of states was limited to two, an LOR was also explicitly calculated. This process was repeated for all cell types and conditions in a comparison (for example, AD versus control).

### Evaluation of the association between splicing and cell state

LOR was used for quantifying the strength of the association between two events, splicing and cell state. For an exon of a specific cell type, we calculated a *P* value, and a Benjamini–Yekutieli correction was performed for multiple testing. For a significant exon, we then used the LOR to quantify the difference in inclusion between both states. Thus, in addition to knowing that the *Ψ* values are significantly different, we can also assess how different they are. The *P* values are derived from the *χ*^2^ test for a 2 × 2 table. Likewise, the LOR is also deduced from the counts of the 2 × 2 table. ‘*A*_inc_’ represents the number of reads that support the exon of a specific cell type in query for state A. ‘*A*_exc_’ represents the number of reads that mapped to the gene but exclude the exon of a specific cell type in query for state A. A similar definition applies to ‘*B*_inc_’ and ‘*B*_exc_*’* for state B.

$${{\mathrm{LOR}}}=\log_2\left(\frac{{A}_{{{\mathrm{inc}}}}/{A}_{{{\mathrm{exc}}}}}{{B}_{{{\mathrm{inc}}}}/{B}_{{{\mathrm{exc}}}}}\right)$$

As described in ‘Long-read data processing’, only spliced reads supporting isoforms observed five or more times per sample (default) were retained in the AllInfo file. We also tested a relaxed cutoff, requiring isoforms to appear five or more times across all samples in a comparison. This led to a modest increase in significantly differentially included exons. Notably, over 80% of exons identified using the strict cutoff were also found with the relaxed cutoff, indicating high consistency. For each cell type in a comparison, only the exons where the total read counts were greater than 10 were retained. Using this, an exon × state matrix of *Ψ* values was obtained per condition, and the matrix for one condition was subtracted from the other, which was defined the as statePSI matrix. To identify the outliers, we limited the statePSI matrix to values that showed at least a 5% difference between conditions and then normalized each row of the statePSI matrix by the RNA-only *Ψ*, thus defining the normState matrix. Finally, in cases where the same exon was tested in both conditions for the same cell type and showed significance in at least one, the state *Ψ* values were plotted against the state to show the divergence in exon inclusion depending on chromatin–RNA state dynamics.

Definitions of *Ψ*, state Δ*Ψ*, overall Δ*Ψ* and normalized-state Δ*Ψ*:

$$\Psi=\frac{{{\rm{inclusion}}\;{\rm{reads}}}}{{{\rm{inclusion}}\;{\rm{reads}}}+{{\rm{exclusion}}\;{\rm{reads}}}}$$

$${\rm{overall}}\,{{\Delta }}\varPsi ={\varPsi }^{{{\mathrm{Case}}}}-{\varPsi }^{{{\mathrm{Ctrl}}}}$$

$${\rm{state}}\,x\,{{\Delta }}\varPsi ={\varPsi }{\rm{(Case}}\,{\rm{state}}\,x{\rm{)}}-{\varPsi }{\rm{(}}{\rm{Ctrl}}\,{\rm{state}}\,x{\rm{)}}$$

$${\rm{normalized}}\; {\rm{state}}\,x\,{{\Delta }}\varPsi =\frac{{\rm{state}}\,x\,\Delta \varPsi }{{\rm{overall}}\,\Delta \varPsi }$$

### Reporting summary

Further information on research design is available in the Nature Portfolio Reporting Summary linked to this article.

## Online content

Any methods, additional references, Nature Portfolio reporting summaries, source data, extended data, supplementary information, acknowledgements, peer review information; details of author contributions and competing interests; and statements of data and code availability are available at 10.1038/s41587-025-02734-5.

## Supplementary information

Supplementary Information Supplementary Figs. 1–20 and Supplementary Table 1.

Reporting Summary

Supplementary Table 2 Sample information of human PFC samples of AD and control groups.

Supplementary Table 3 List of significant brain-region-specific exons in L3–L5/L6 IT_*RORB* excitatory neurons of macaques.

Supplementary Table 4 List of significant brain-region-specific exons in L2–L4 IT_*CUX2.RORB* excitatory neurons of macaques.

Supplementary Table 5 List of significant brain-region-specific exons in L2–L3 IT_*CUX2* excitatory neurons of macaques.

Supplementary Table 6 List of significant AD-specific exons across all major cell types in the human PFC.

## Data Availability

The human and macaque short-read and long-read datasets used in this study are available at https://www.ncbi.nlm.nih.gov/sra/PRJNA1021558 (ref. ^99^). All the data used to support the findings of this study are provided within the paper and are publicly available at https://www.gencodegenes.org/human (ref. ^78^), https://ftp.ensembl.org/pub/release-104/gtf/mus_musculus/ (ref. ^79^), https://www.blueprintnhpatlas.org (ref. ^36^), https://azimuth.hubmapconsortium.org/references/human_motorcortex/ (refs. ^37,82^) and https://compbio.mit.edu/ad_aging_brain/ (ref. ^83^).

## Code availability

The package scisorATAC is available at https://github.com/careenfoord/scisorATAC (ref. ^100^).

Other analysis pipelines are available at https://github.com/wenhu0701/ScisorATAC (ref. ^101^).
