# Supplementary material for: Combined single-cell profiling of chromatin–transcriptome and splicing across brain cell types, regions and disease state
Source: Nat Biotechnol. 2025 Jul 22;44(6):976–88. doi: 10.1038/s41587-025-02734-5 (PMC12425497; doi:10.1038/s41587-025-02734-5)
Supplement: Supplementary file 1 — Supplementary Figs. 1–20 and Supplementary Table 1. [file 41587_2025_2734_MOESM1_ESM.pdf]

nature biotechnology

Supplementary information https://doi.org/10.1038/s41587-025-02734-5

Combined single-cell profiling of chromatin–transcriptome and splicing across brain cell types, regions and disease state

In the format provided by the authors and unedited

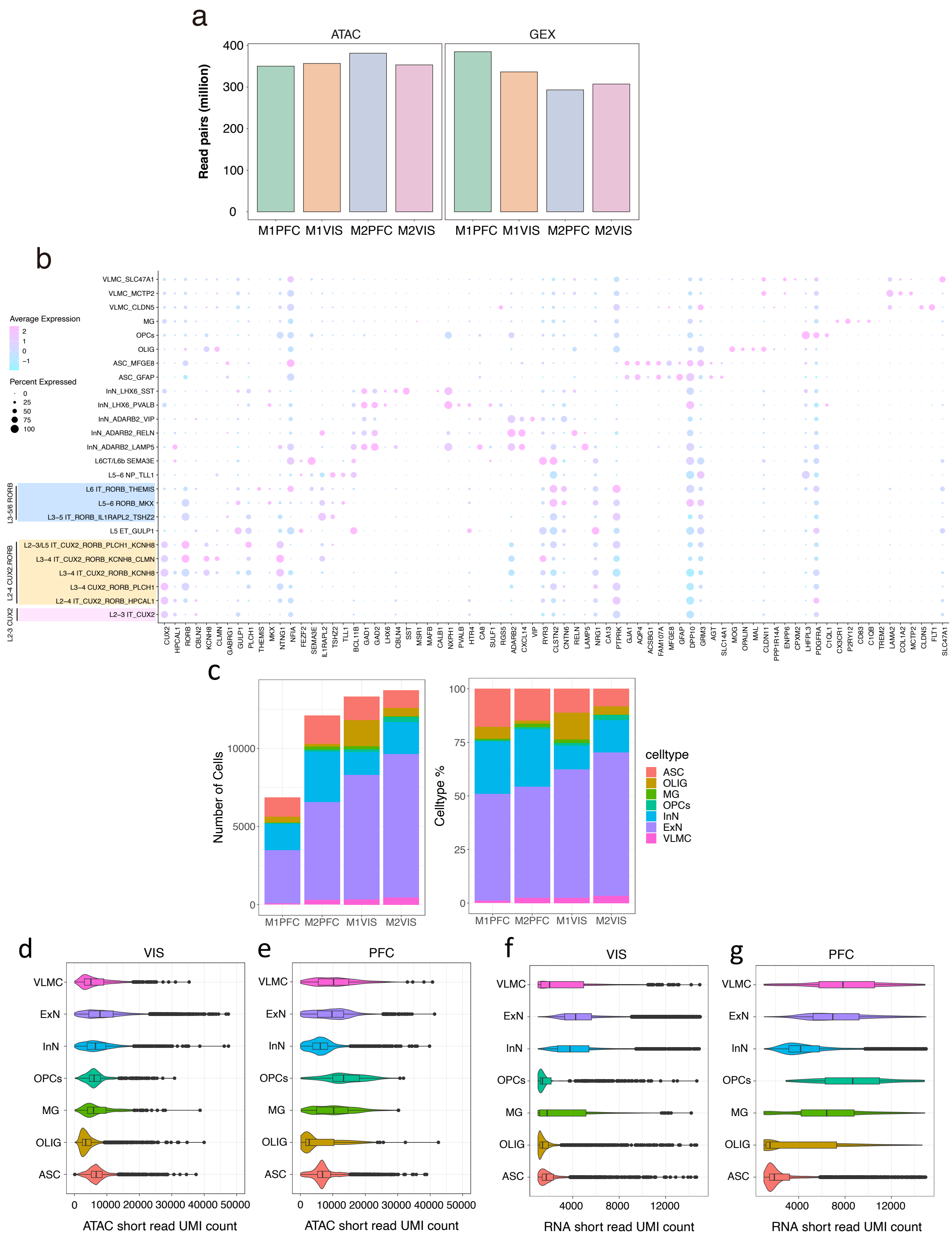

Supplementary Fig. 1. **a**, Number of read pairs for each sample for both ATAC and RNA sequencing. **b**, Dot plot of marker genes expressed across defined cell types and subtypes. **c**, Number and percent of all the major cell types annotated for PFC and VIS samples. **d-e**, Number of unique UMI counts of each cell type and subtype within ATAC data. **f-g**, Number of unique UMI counts of each cell type and subtype within RNA data. OLIG, oligodendrocyte; MG, microglia; InN, inhibitory neurons; ASC, astrocyte; ExN, excitatory neurons; VLMC, vascular and leptomeningeal cells; OPCs, oligodendrocyte precursor cells. Each boxplot shows the median (middle line), interquartile range (IQR, top and bottom of the box) and whiskers extending to 1.5× IQR. Dots represent outliers.

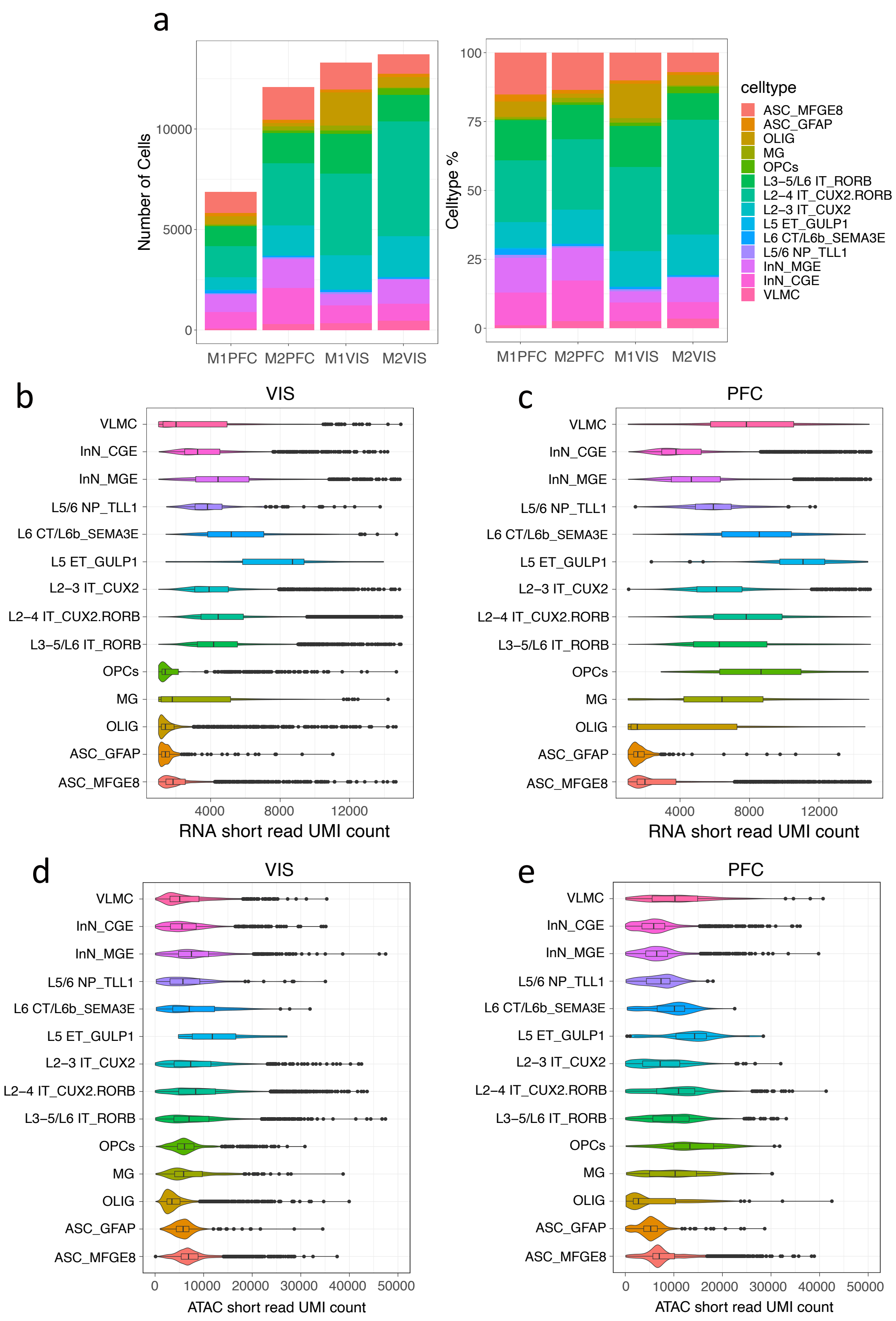

Supplementary Fig. 2. a, Number and percent of cells of subtypes per macaque sample. **b-c**, UMI counts revealed by RNA short reads of each subtype per brain region (PFC and visual cortex) of macaque samples. **d-e**, UMI counts revealed by ATAC short reads of each subtype per brain region (PFC and visual cortex) of macaque samples. Same cell type nomenclature as described in Supplementary Fig. 1. Each boxplot shows the median(middle line of the box), interquartile range (IQR, top and bottom of the box) and whiskers extending to 1.5× IQR. Dots represent outliers.

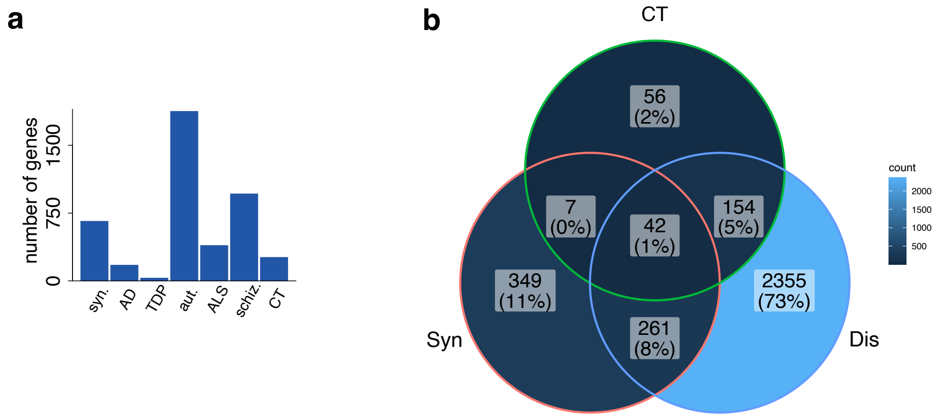

Supplementary Figure 3. **a,** Number of genes per category in macaque for customized Agilent enrichment probes targeting exon-exon junctions (Methods). Groups on the x-axis include synaptic (syn), Alzheimer's Disease (AD), TDP43-affected (TDP), Autism (aut), Amyotrophic Lateral Sclerosis (ALS), Schizophrenia (schiz), or cell type specific (CT) genes. **b,** Venn Diagram indicating number and % of overlapping genes across groups in macaque.

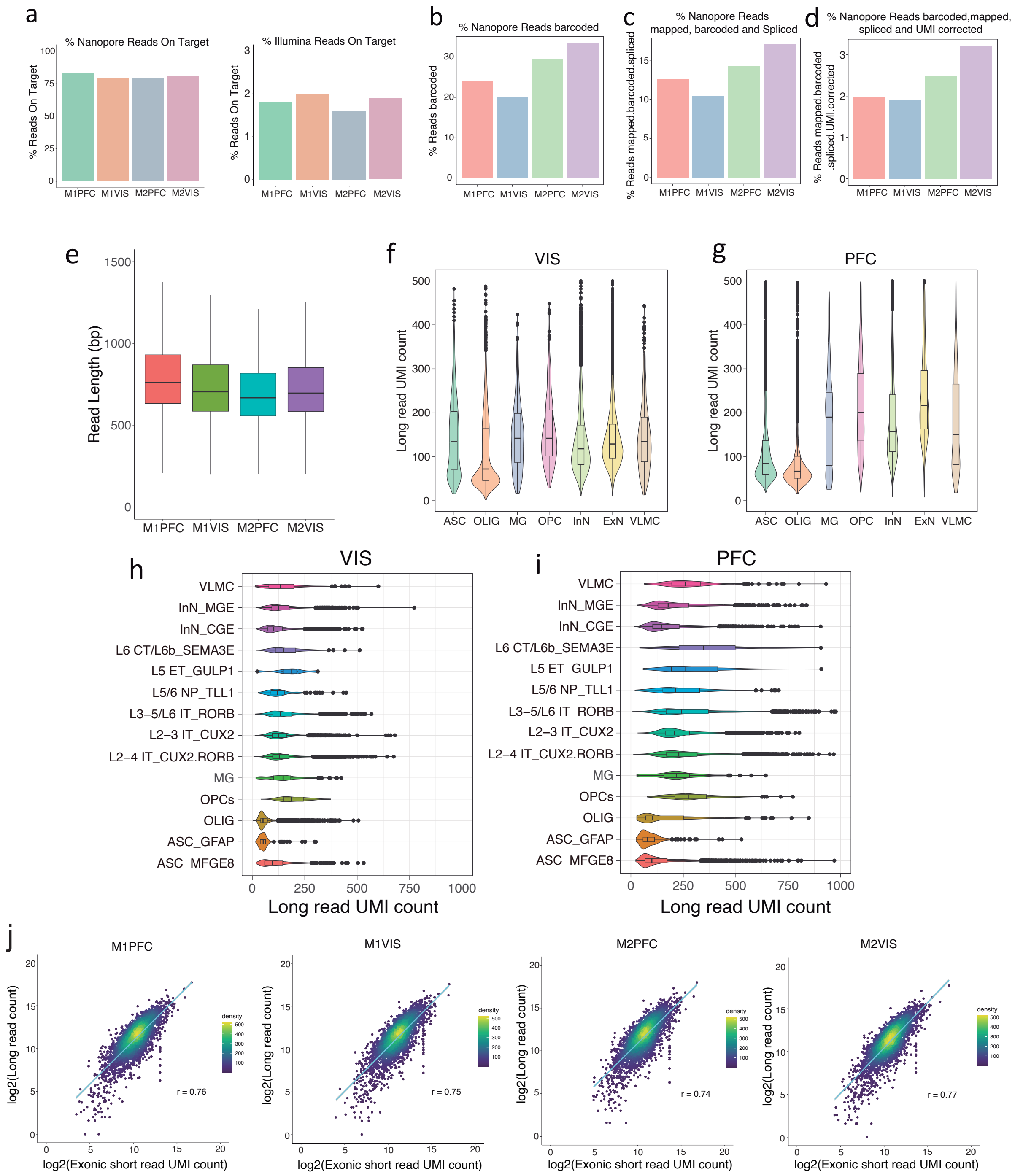

Supplementary Fig. 4. Percentage of on-target reads among **a**, long reads and RNA short reads per macaque sample. **b**, Percentage of mapped and barcoded reads among long reads per sample. **c**, Percentage of mapped/barcoded/spliced reads among long reads per sample. **d,** Percentage of mapped/barcoded/spliced/UMI corrected reads among long reads per sample. **e**, 10,000 long reads per sample were subsampled for showing the read length distribution of macaque PFC and VIS samples (n=10,000). **f-g**, UMI counts revealed by long reads per major cell type of each brain region (PFC and Visual cortex). Same cell type nomenclature as described in Supplementary Fig. 1. **h-i**, UMI counts revealed by long reads per subtype of each brain. **j**, Correlation between UMI counts of exonic short reads (log2 transformed) and exonic long reads per sample (log2 transformed). The regression line was plotted using the linear model. Each boxplot shows the median (middle line of the box), interquartile range (IQR, top and bottom of the box) and whiskers extending to 1.5× IQR. Dots represent outliers.

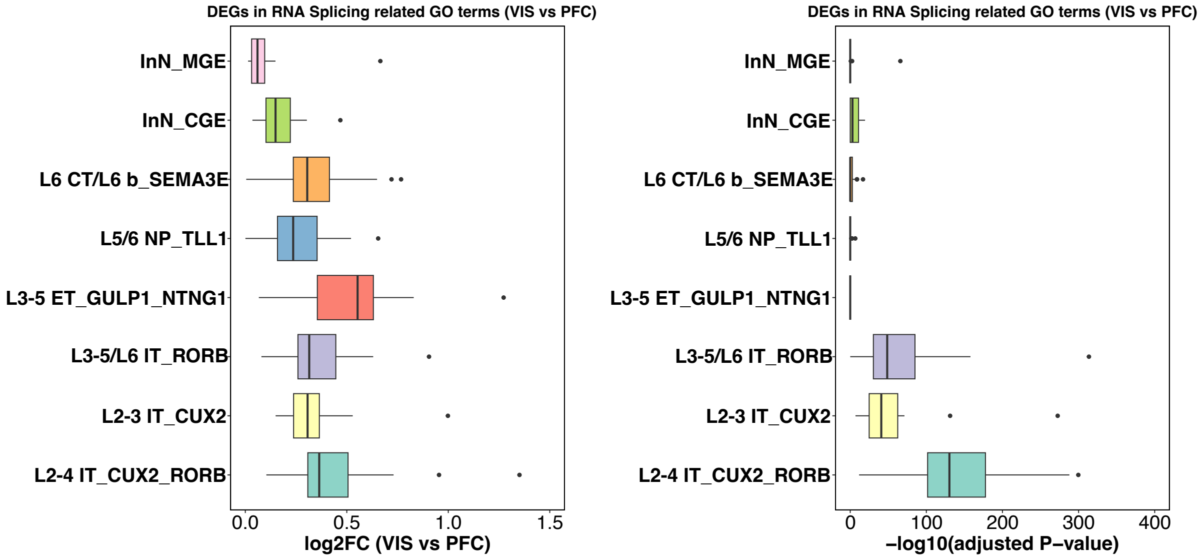

Supplementary Fig. 5. DEGs (n=17) included in the RNA splicing related GO terms (GO:0000375/GO:0000377/GO:0000398) showed more dramatic fold changes in excitatory neurons than inhibitory neurons. The log2-fold change values and corresponding adjusted P-values are shown per cell-type. Each boxplot shows the median (middle line of the box), interquartile range (IQR, top and bottom of the box) and whiskers extending to 1.5× IQR. Dots represent outliers.

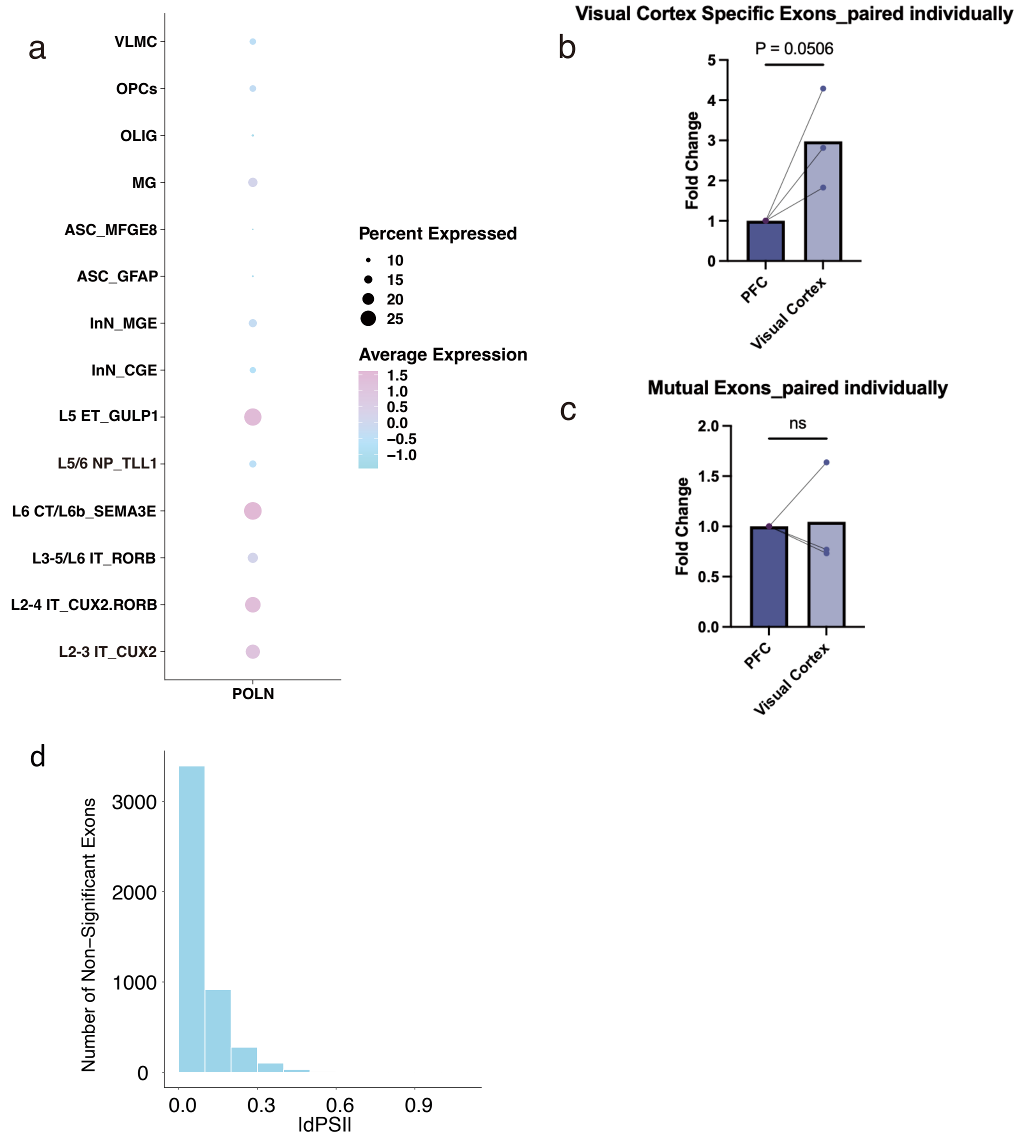

e

Reads per condition (PFC vs. VIS)	|deltaPSI|				
	0.1	0.2	0.3	0.4	0.5
50-249	21	70	84	98	100
40-49	0	18	68	91	97
30-39	0	5	45	82	96
20-29	0	0	11	53	75
10-19	0	0	1	17	38
0-9	0	0	0	0	0

Supplementary Fig. 6. **a**, Plot indicating average expression of POLN and percent of cells expressed in across defined cell types and subtypes. **b**, Fold change of POLN exons chr5:2192426-2192544(-) and chr5:2233840-2233894(-) in PFC and Visual Cortex measured via QPCR(n = 3). **c**, Fold change of mutual control POLN exons in Prefrontal Cortex (PFC) and Visual Cortex measured via QPCR(n = 3). **d**, Number of non-significant exons within each $|\Delta\Psi|$ bin (from 0 to 1, bin = 0.1). **e**, The fraction of matrices that pass Benjamini-Yekutieli correction for multiple testing to find a $\Delta\Psi$ of 0.1, 0.2, 0.3, 0.4 and 0.5 by downsampling the combined read counts of [0,9], [10,19], [20,29], [30,39], [40,49], [50,249] per condition.

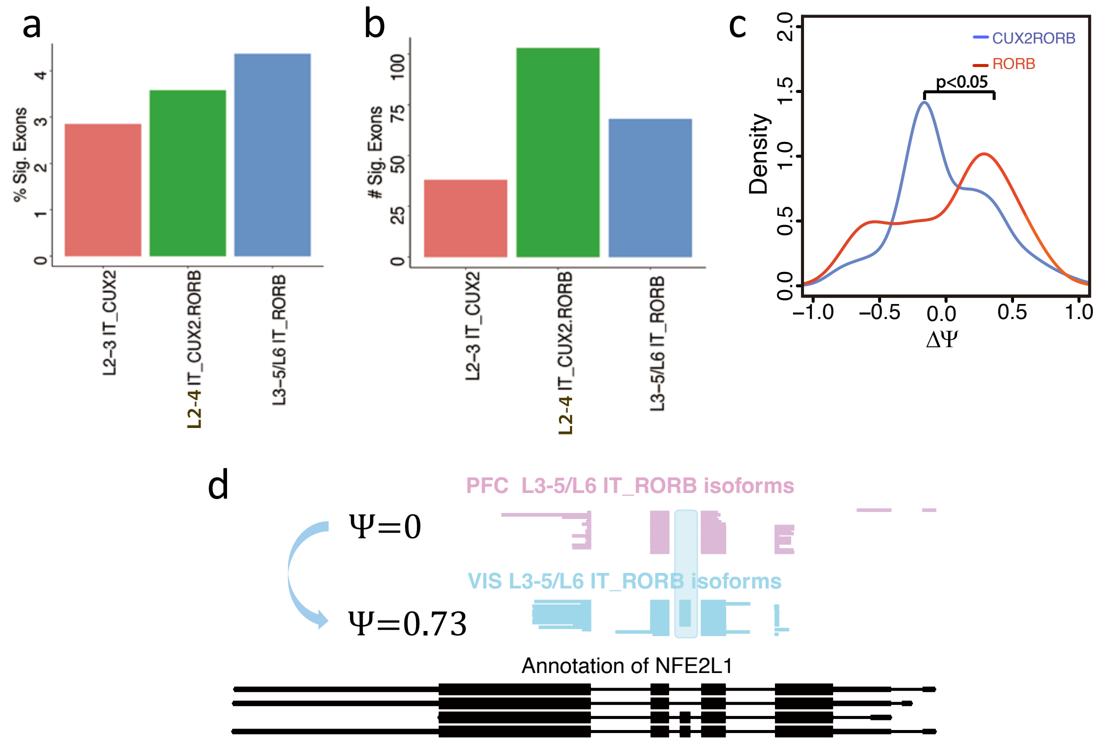

Supplementary Fig. 7. **a,** Percentage of exons that are significantly different utilized between PFC and visual cortex for each excitatory neuron subtype. **b,** Number of exons that are significantly different utilized between PFC and visual cortex for each excitatory neuron subtype. **c**, Density plot of the $\boldsymbol{\Delta\Psi}$ (PFC-VIS) of L3-5/L6 IT_*RORB and* L2-4 IT_*CUX2.RORB* excitatory neuron (number of tested exons of two subtypes: n = 1,558 and 2,881 respectively). Two-sided Wilcoxon-rank-sum test was applied to the comparison ($p < 0.05$). **d**, Cell-type resolved single-cell long reads for NFE2L1 gene plotted with ScISOrWiz. Top 2 tracks: L3-5/L6 IT_*RORB* in PFC and VIS. Bottom black track: chr16:48,747,115-48,754,073.

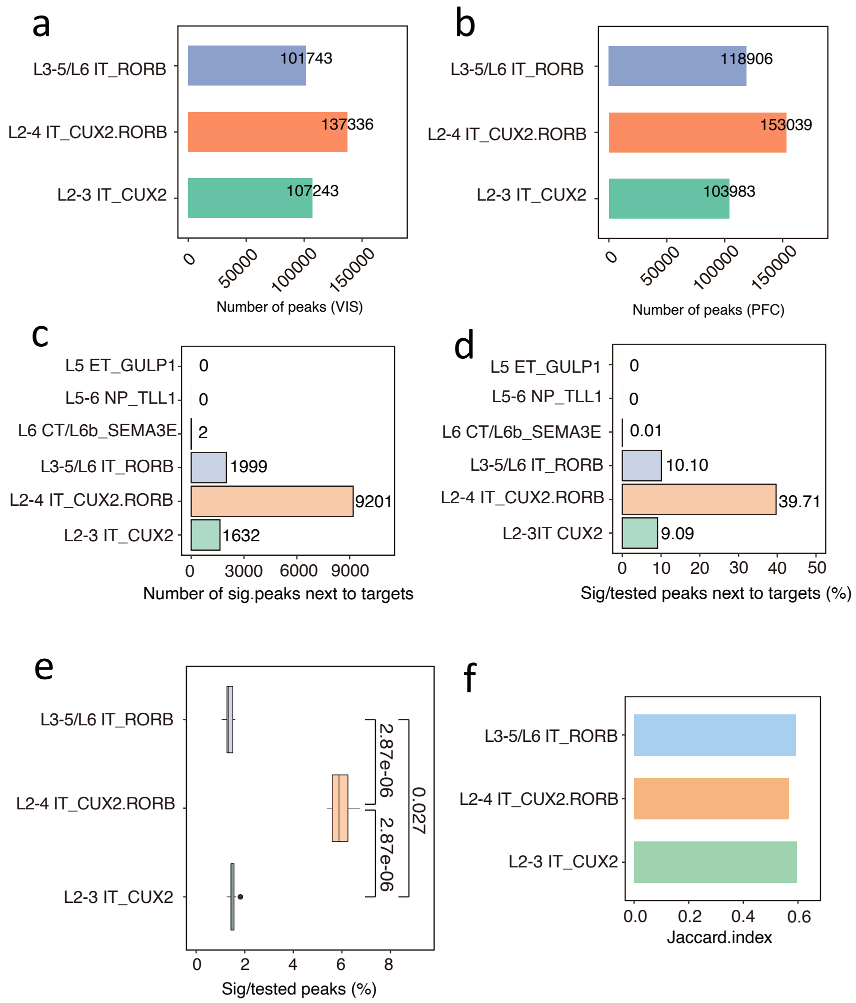

Supplementary Fig. 8. **a**, Number of visual cortex peaks defined for excitatory subtypes L2-3 IT_*CUX2*, L3-5/L6 IT_*RORB*, and L2-4 IT_*CUX2.RORB*. **b**, Number of PFC peaks defined for excitatory subtypes L2-3 IT_*CUX2*, L3-5/L6 IT_*RORB*, and L2-4 IT_*CUX2.RORB*. **c**, Number of peaks that are significantly different for each type of excitatory neuron in the vicinity of genes targeted for splicing analysis. **d**, Percentage of peaks that are significantly different for each type of excitatory neuron in the vicinity of genes targeted for splicing analysis. **e**, Differential accessibility between conditions (VIS/PFC) evaluated by random subsampling of peaks. With the cell type predicted based on the transferred labels from RNA to ATAC data, 10,000 peaks were randomly selected among all the peaks called from random subsampled 1,000 cells of VIS/PFC samples for each excitatory neuron subtype. The random sampling was repeated for 20 times (n=20). The ratio of significant peaks closest to the target genes were shown. Each boxplot shows the median (middle line), interquartile range (IQR, top and bottom of the box) and whiskers extending to 1.5× IQR. Dots represent outliers. FDR adjusted two-sided Wilcoxon-rank-sum test p-values were shown for each comparison. **f**, Jaccard index for overlap of peaks between the two brain regions for each type of excitatory neurons, among which L2-4 IT_CUX2.RORB shows the lowest similarity.

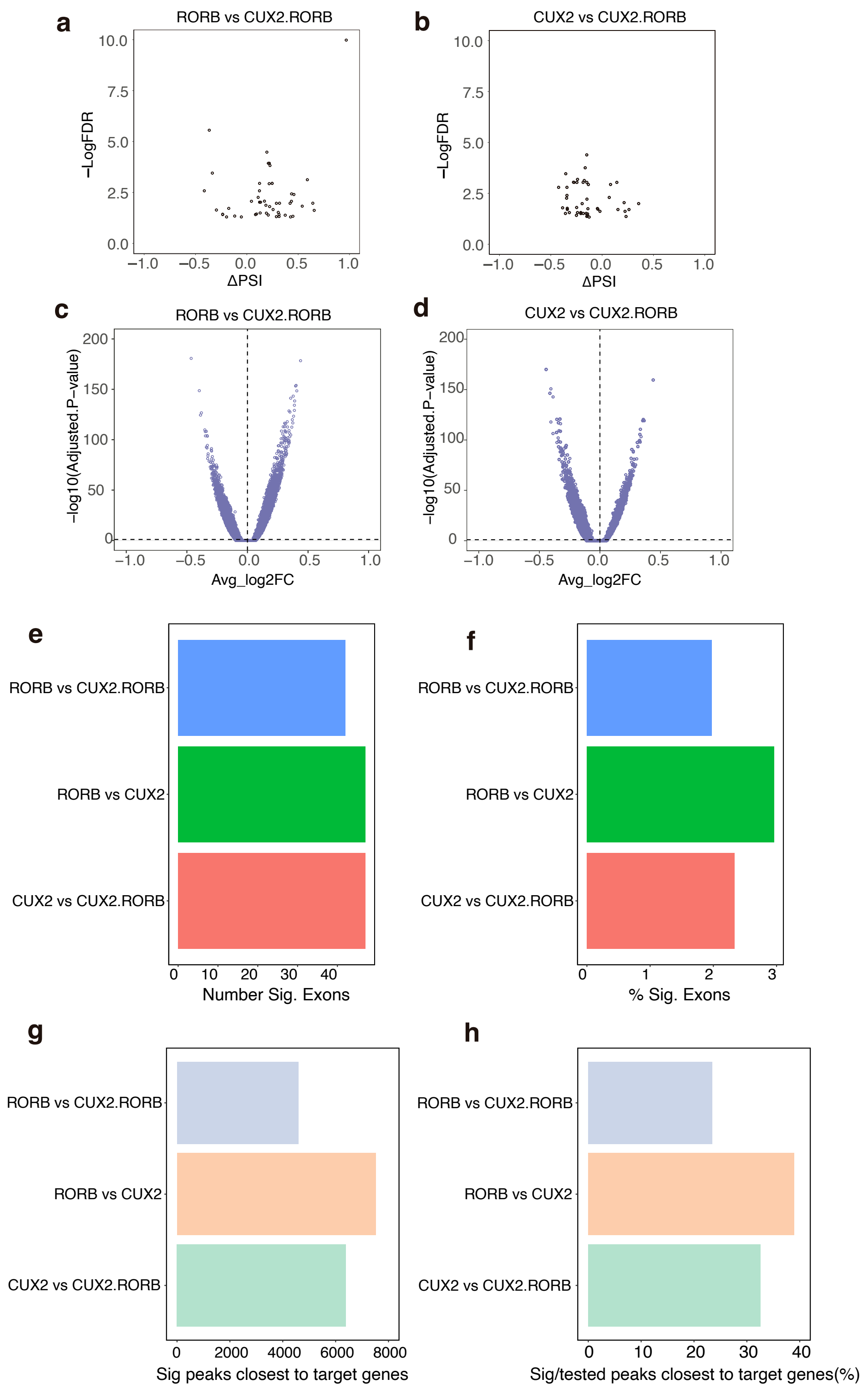

Supplementary Fig. 9. **a**, Volcano plot of differentially spliced exons between RORB and CUX2.RORB excitatory subtypes in both brain areas combined. **b,** Differentially spliced exons between CUX2 and CUX2RORB excitatory subtypes. **c,** Differential accessible open chromatin regions between RORB and CUX2.RORB. **d,** Differential accessible different open chromatin regions between CUX2 and CUX2.RORB. (CUX2 represents L2-3 IT_*CUX2*, RORB represents L3-5/L6 IT_*RORB*, and CUX2.RORB represents L2-4 IT_*CUX2.RORB*). **e-f**, Number and percentage of significant differentially spliced exons in three excitatory subtype comparisons. **g-h**, Number and percentage of significant peaks closest to the targeted genes in three excitatory subtype comparisons.

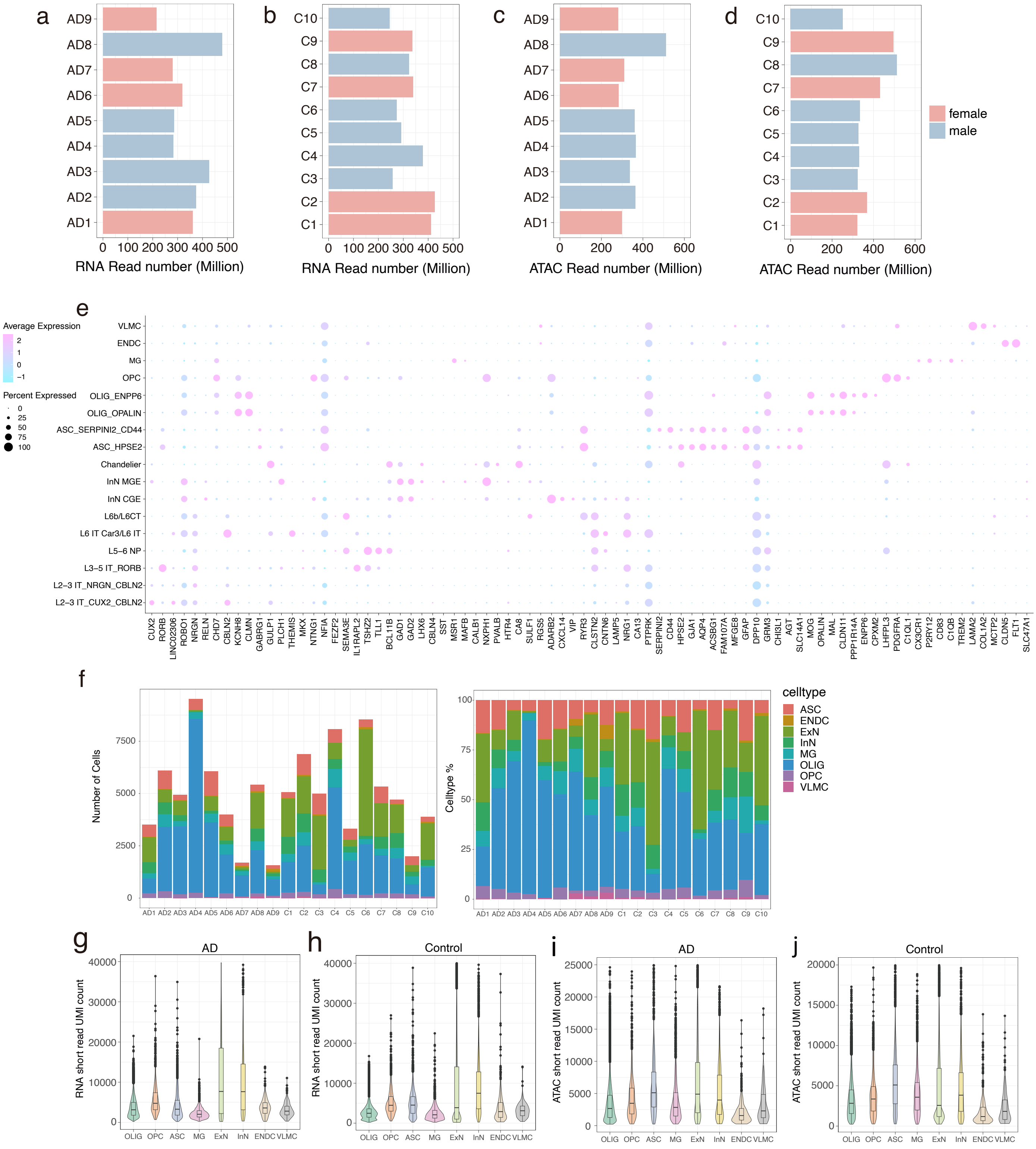

Supplementary Fig. 10. **a-d**, Number of RNA/ATAC read pairs per sample. **e**, Dotplot of cell type markers. **f**, Number/percentage of cells per major cell type. **g-j**, Violin/boxplot of RNA/ATAC UMI counts per major cell type per condition (AD and control samples). Same cell type nomenclature as described in Supplementary Fig. 1. Each boxplot shows the median (middle line of the box), interquartile range (IQR, top and bottom line of the box) and whiskers extending to 1.5× IQR. Dots represent outliers beyond this range.

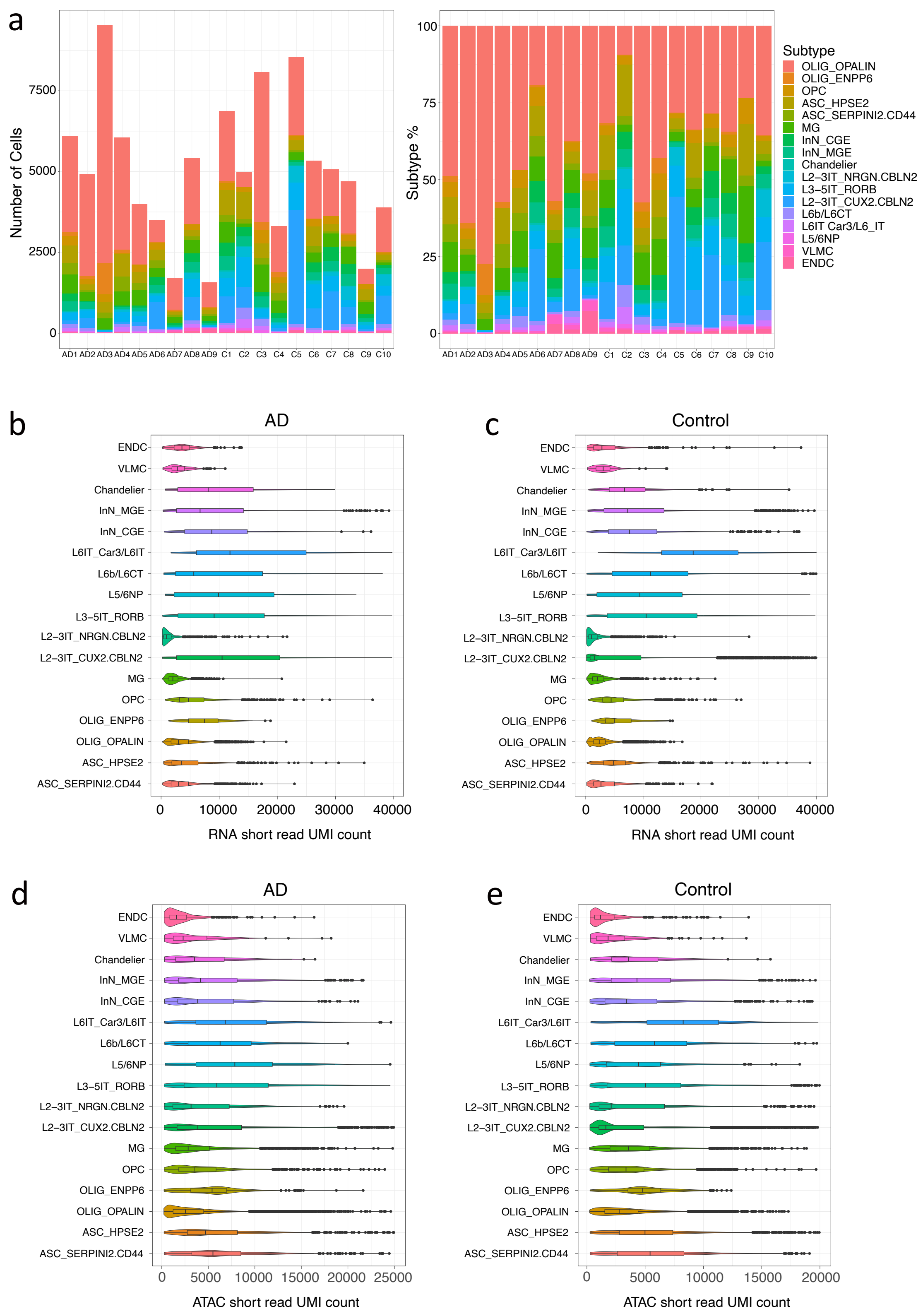

Supplementary Fig. 11. **a**, Number/proportion of cells per major cell type. **b-e**, Violin/boxplot of RNA/ATAC UMI counts per subtype per condition (AD and control). Same cell type nomenclature as described in Supplementary Fig. 1. Each boxplot shows the median (middle line of the box), interquartile range (IQR, top and bottom line of the box) and whiskers extending to 1.5× IQR. Dots represent outliers.

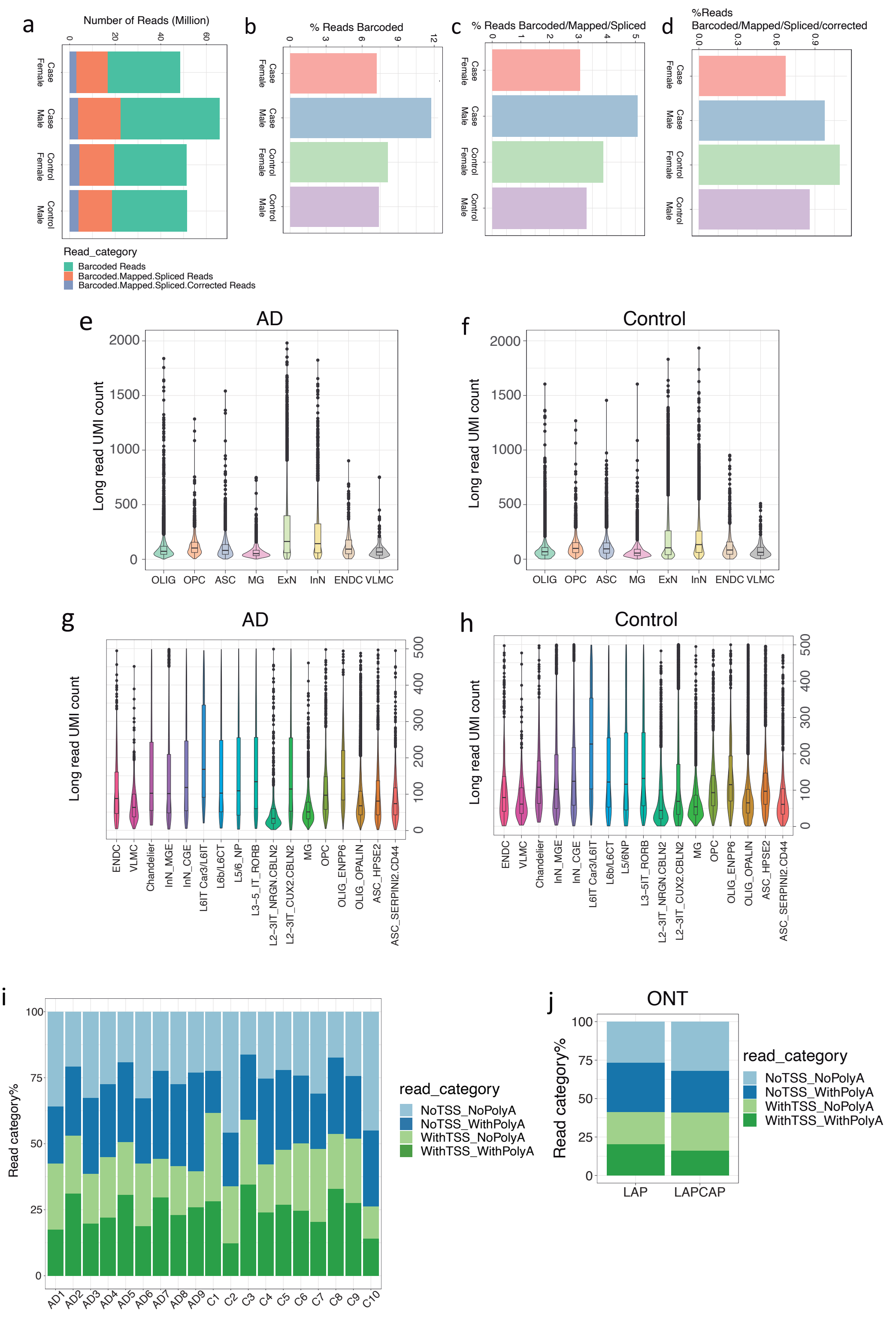

Supplementary Fig. 12. **a-d**, Number/percentage of barcoded reads/barcoded, mapped and spliced reads/barcoded, mapped, spliced and corrected reads per condition (AD, control, male, female). **e-f**, Long read UMI counts per condition per major cell-type. **g-h**, Long read UMI counts per condition per subtype. **i**, Completeness of long reads of 19 human PFC samples derived from exome enrichment targeting 3,631 brain genes by junction probes. **j**, Completeness of two human PFC samples' long reads with/without exome enrichment. Same cell type nomenclature as described in Supplementary Fig. 1. Each boxplot shows the median (middle line of the box), interquartile range (IQR, top and bottom line of the box) and whiskers extending to 1.5× IQR. Dots represent outliers.

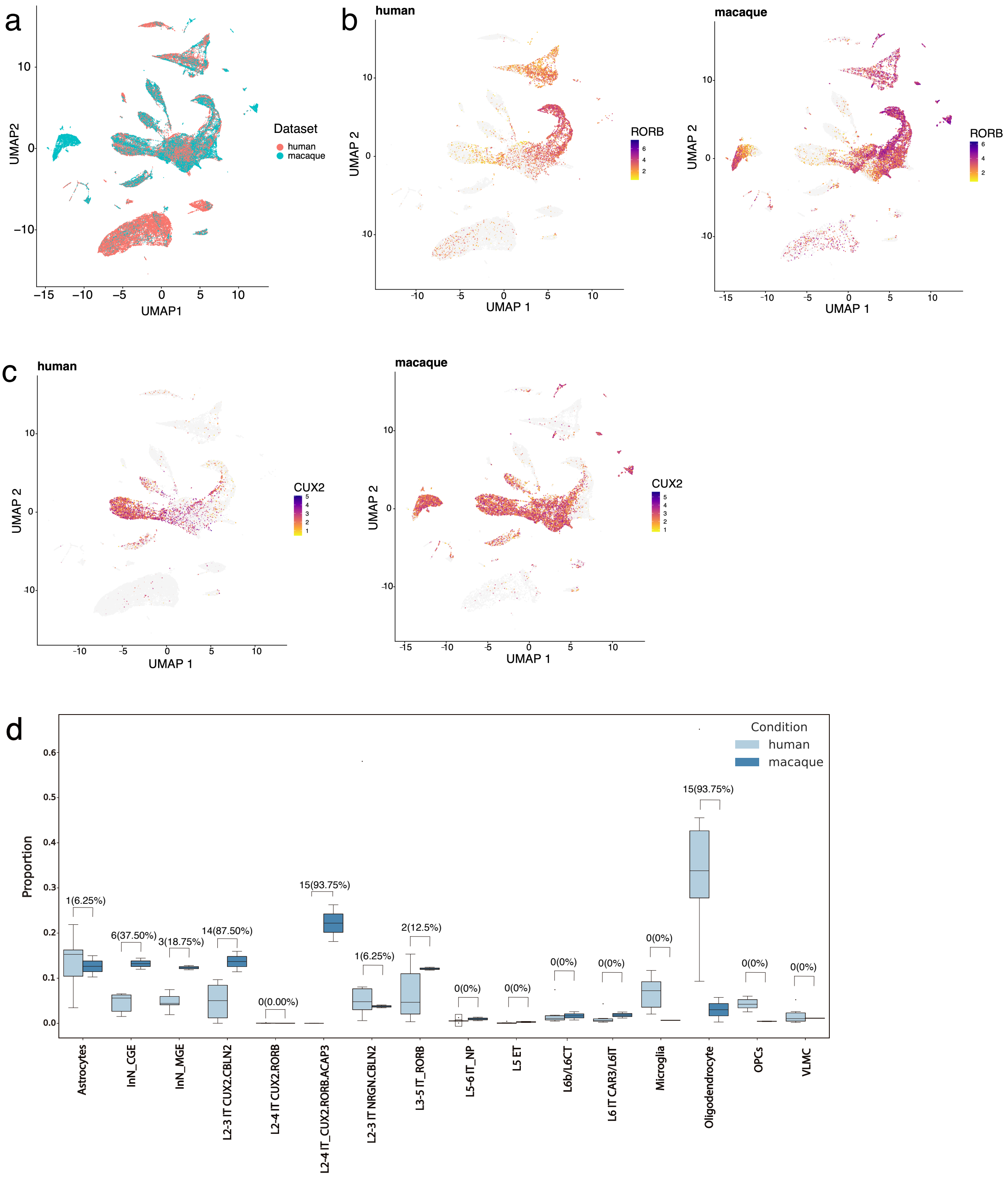

Supplementary Fig. 13. **a,** UMAP of human control PFC and macaque PFC samples. **b,** Expression of *RORB* in human control PFC and macaque PFC samples. **c,** Expression of *CUX2* in human control PFC and macaque PFC samples. **d,** Cell-type composition in two species. Numbers on top of the boxes indicate: the frequency and the percentage of all iterations which shows the credible cell-type composition difference between species by sequentially selecting each cell type as the reference once (n=16). The composition of three cell types (L2-3 IT_*CUX2.CBLN2*, L2-4 IT_*CUX2.RORB.ACAP3* and oligodendrocytes) are found to be different between two species in >80% of the iterations, whereas >50% is considered to be credible. Each boxplot shows the median (middle line of the box), interquartile range (IQR, top and bottom line of the box) and whiskers extending to 1.5× IQR.

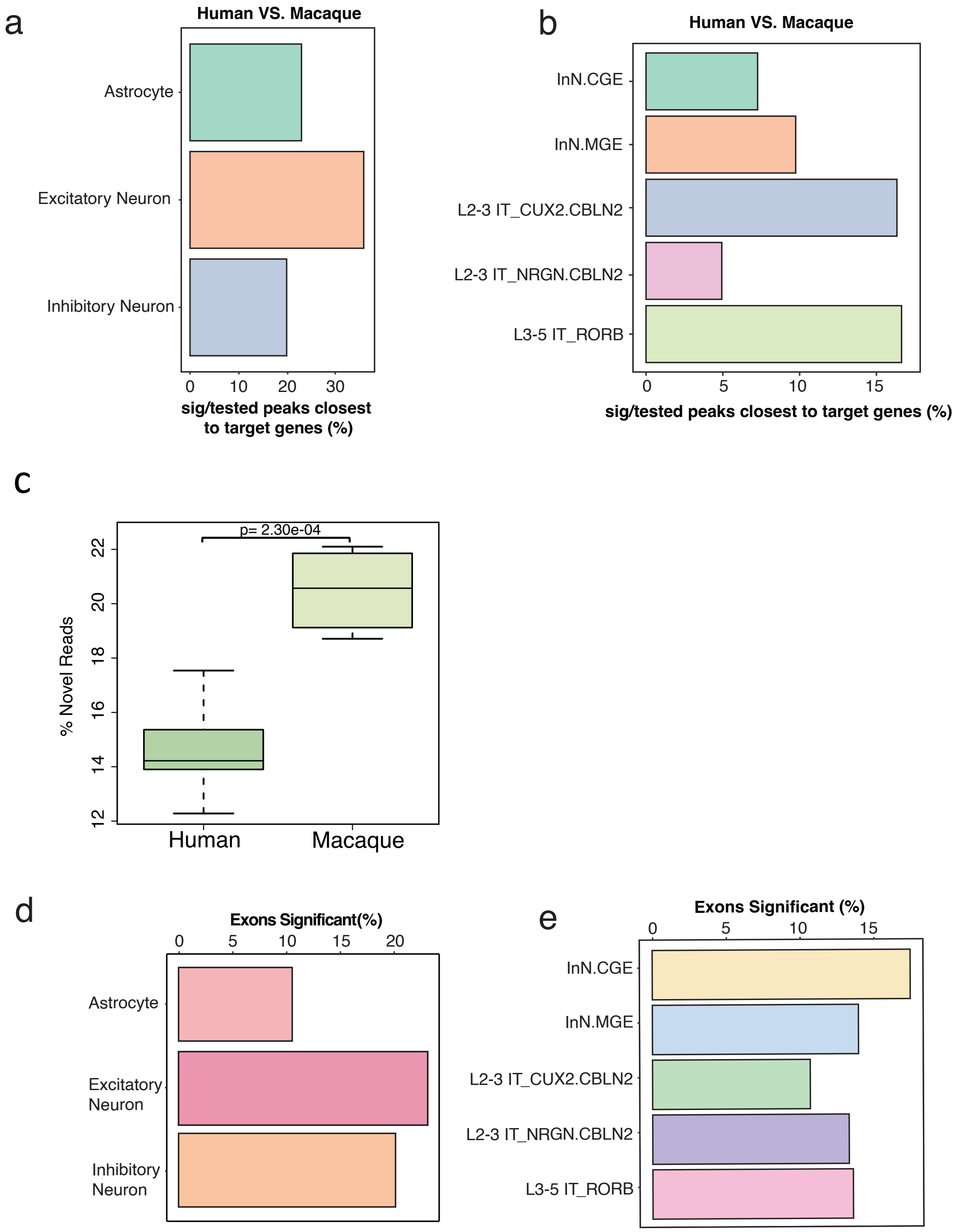

Supplementary Fig. 14. **a**, Percent significant peaks close to target genes tested in inhibitory and excitatory neurons, and astrocytes. **b**, Percent significant peaks close to target genes tested in excitatory and inhibitory neuron subtypes. **c**, Percent of reads support novel isoforms in human (n=6) and macaque (n=2) PFC datasets, two-sided Wilcoxon-rank-sum test p-value =2.3e-06. **d**, Percent exons significant between species in inhibitory and excitatory neurons, and astrocytes. **e**, Percent exons with significant splicing differences between species in excitatory and inhibitory neuron subtypes. Each boxplot shows the median (middle line of the box), interquartile range (IQR, top and bottom line of the box) and whiskers extending to 1.5× IQR.

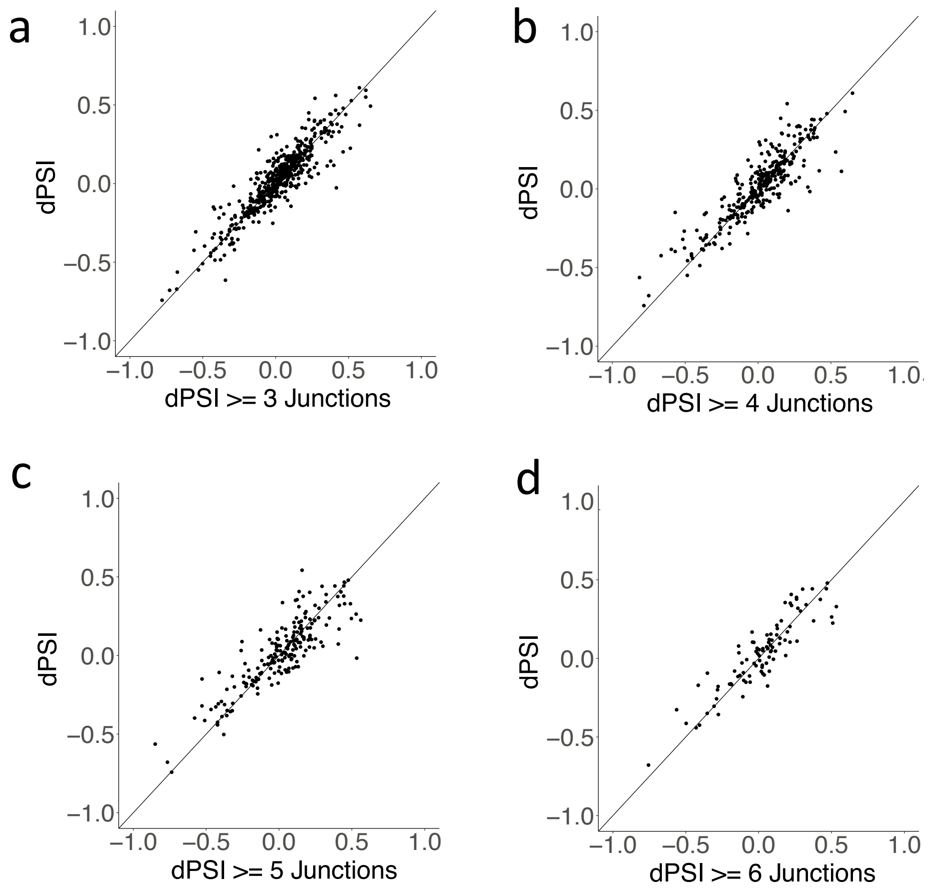

Supplementary Fig. 15. Correlation between exon dPSI($\boldsymbol{\Delta\Psi}$) revealed by all reads (y axis) and reads with **a** >=3 or **b** >=4 or **c** >= 5 or **d** >=6 junctions. The regression line was generated using the linear regression model.

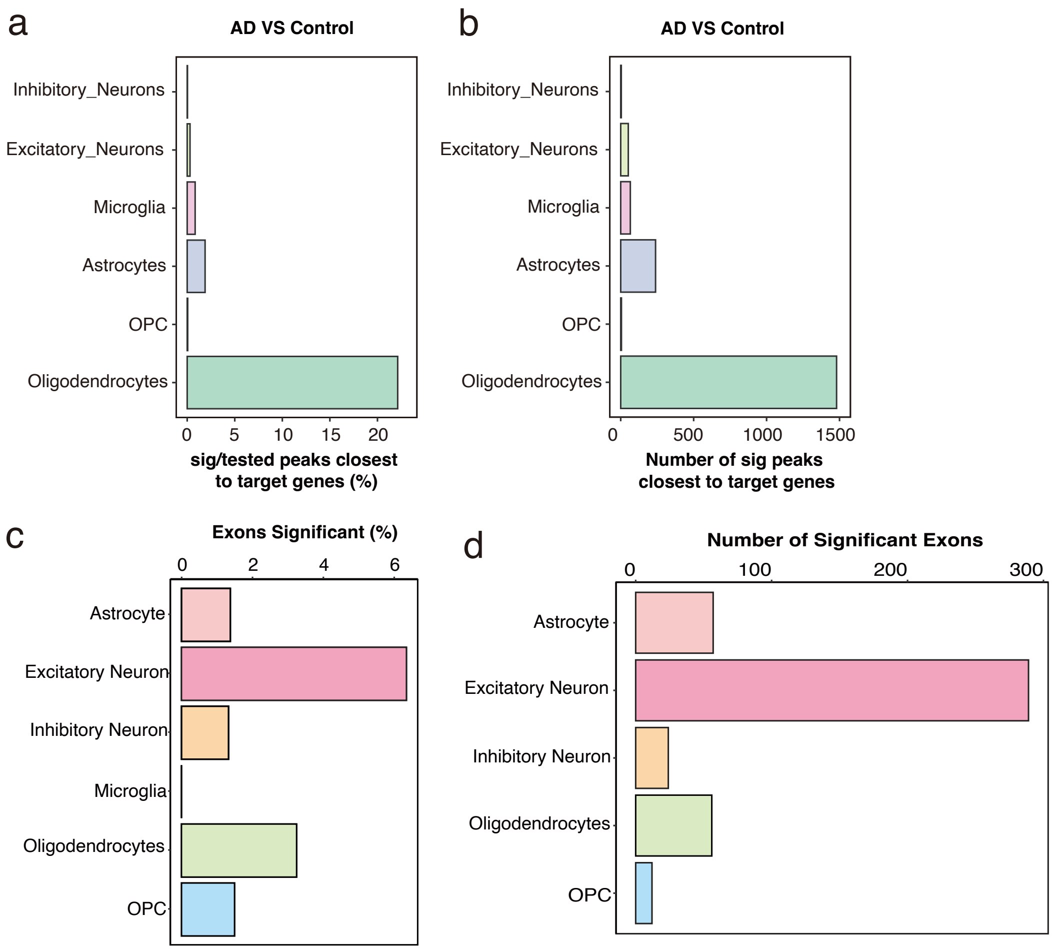

Supplementary Fig. 16. **a-b**, Percent and number of significant peaks out of peaks close to target genes tested in inhibitory neurons, excitatory neurons, microglia, astrocytes, OPCs and oligodendrocytes. **c-d**, Percent and number of exons significant between AD and control in astrocytes, excitatory neurons, inhibitory neurons, oligodendrocytes and OPCs (**Supplementary Table 6**).

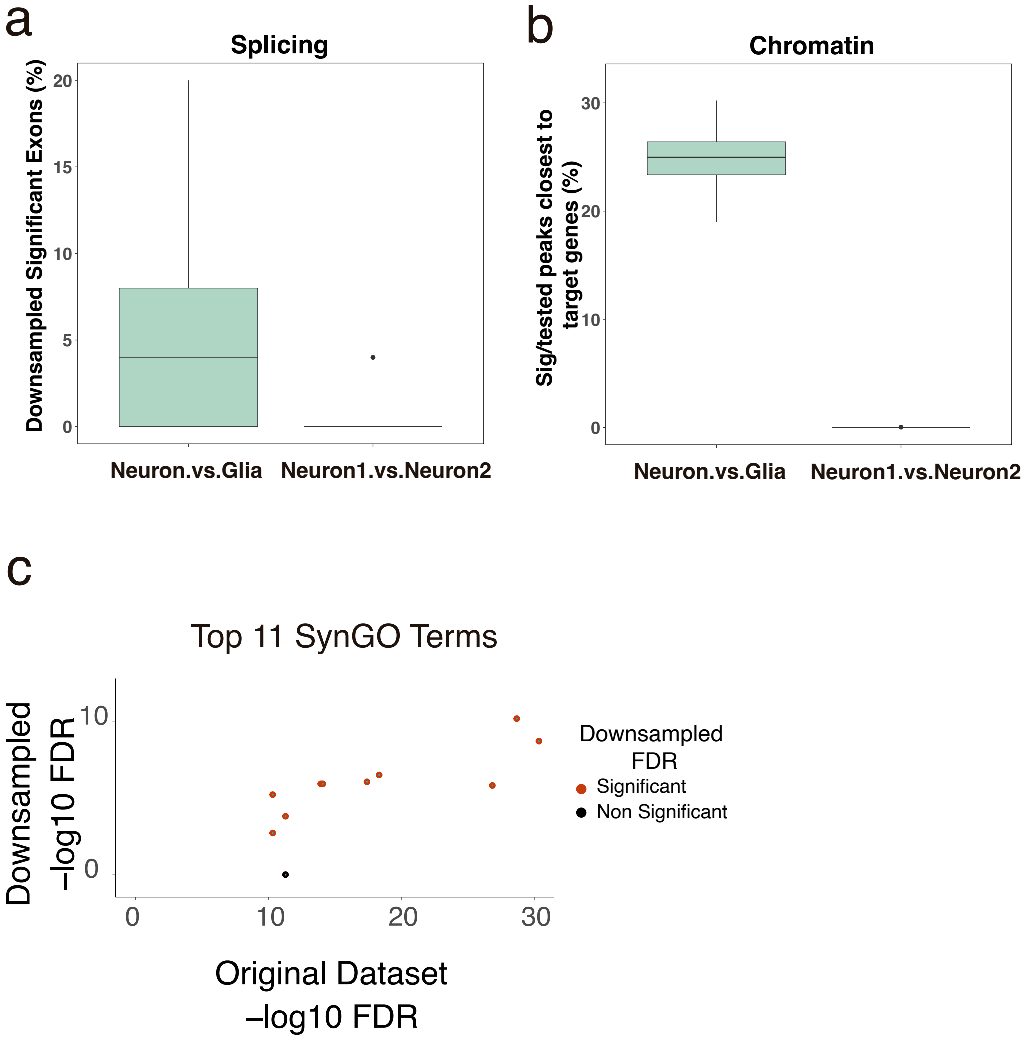

Supplementary Fig. 17. **a**, After performing100 times downsampling, the boxplot shows the percent of significant dysregulated exons between neuron cells and glia cells, also between neuron group1 and neuron group2 which are derived from randomly splitting the all neurons into two groups (n=100 per group). **b**, After performing100 times downsampling, the boxplot shows the percent of significant peaks with differential accessibility between neuron cells and glia cells, also between neuron group1 and neuron group2 described in a (n=100). **c**, the -log10(FDR) of top 11 SynGO terms shared by both SynGO analysis based on the genes with significant differential exon-inclusion in either original dataset or union of 100 times downsampling. Each boxplot shows the median (middle line of the box), interquartile range (IQR, top and bottom line of the box) and whiskers extending to 1.5× IQR. Comparisons presented in **a** and **b** both showed a p-value<2.2e-16 using two-sided Wilcoxon-rank-sum test.

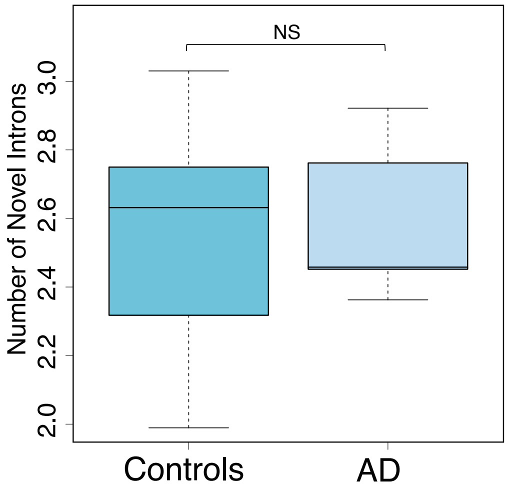

Supplementary Fig. 18. Number of novel introns in Control (n=10) and AD (n=9) samples. Each boxplot shows the median (middle line of the box), interquartile range (IQR, top and bottom line of the box) and whiskers extending to 1.5× IQR. The comparison was performed using two-sided Wilcoxon-rank-sum test.

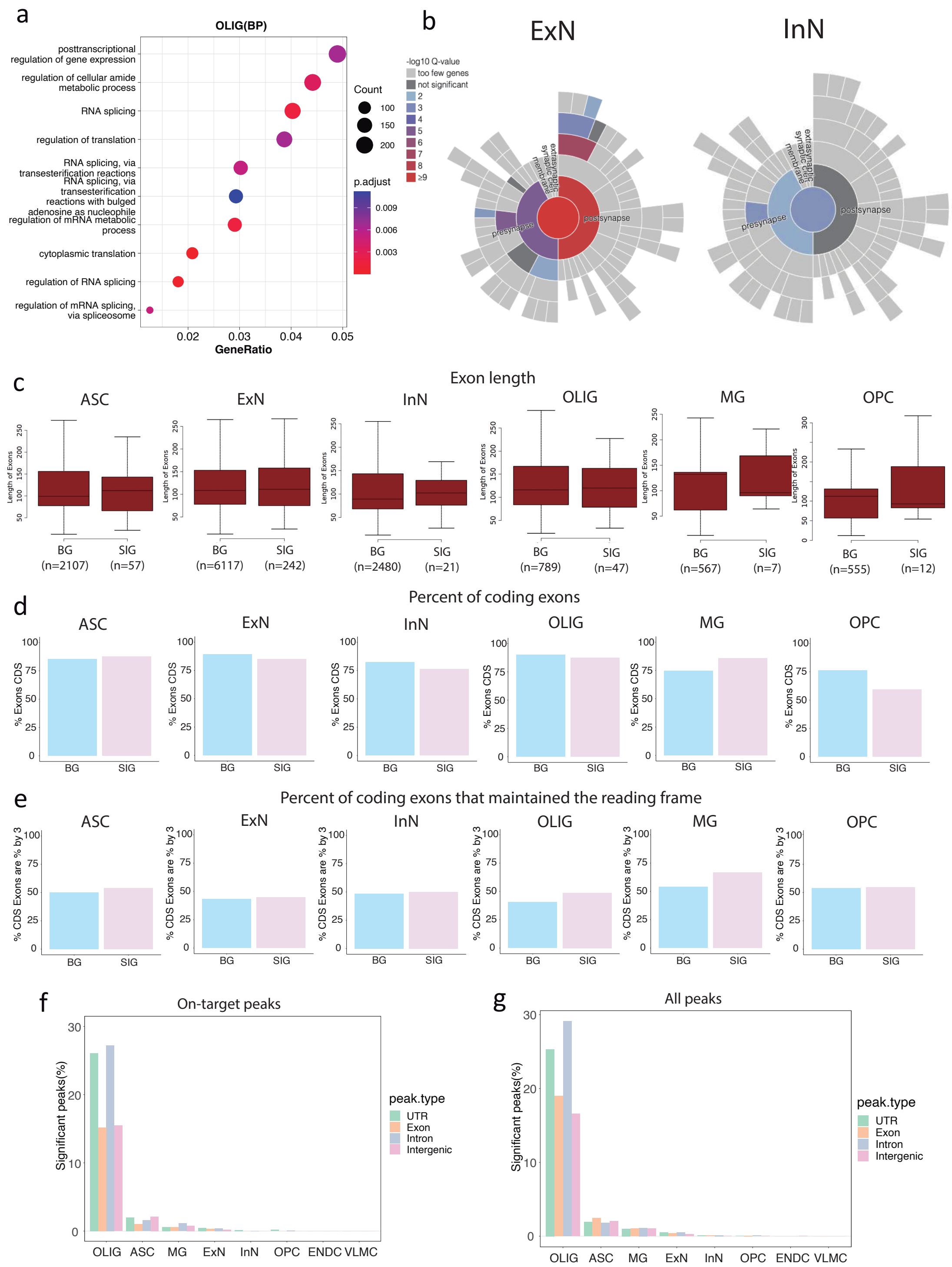

Supplementary Fig. 19. **a**, GO enrichment (Biological process) of genes have significant differential accessible peaks covered by comparing AD and controls PFC samples. **b**, SynGO analysis of genes with splicing changes in excitatory neurons and inhibitory neurons between Alzheimer cases and controls by using the genes with tested but non-significant exons as a background. **c**, Length of significant and non-significant exons in major cell types. **d**, Percent of significant and non-significant exons in major cell types. **e**, Percent of coding exons that maintained the reading frame in major cell types. **f**, Percent of significant on-target peaks per peak category (UTR/Exon/Intron/Intergenic peaks). **g**, Percent of significant peaks per peak category (UTR/Exon/Intron/Intergenic peaks). Each boxplot shows the median (middle line of the box), interquartile range (IQR, top and bottom line of the box) and whiskers extending to 1.5× IQR.

a

chr3_108050578_108050602_ENSG00000196776.16_- (CD47)

State 1	AD	Ctrl
Inclusion	1	NA
Exclusion	0	NA
Ψ(PSI)	1	NA

State1 $\Delta\Psi$ = NA
normalized state1 $\Delta\Psi$ = NA

State 2	AD	Ctrl
Inclusion	297	182
Exclusion	50	66
Ψ(PSI)	0.856	0.734

State2 $\Delta\Psi$ = 0.122
normalized state2 $\Delta\Psi$ = 1.038

State 3	AD	Ctrl
Inclusion	15	244
Exclusion	0	80
Ψ(PSI)	1	0.753

State3 $\Delta\Psi$ = 0.247
normalized state3 $\Delta\Psi$ = 2.102

Overall	AD	Ctrl
Inclusion	313	426
Exclusion	50	146
Ψ(PSI)	0.862	0.745

Overall $\Delta\Psi$ = 0.117

b

chr5_132708903_132708978_ENSG00000131437.15_- (KIF3A)

State 2	AD	Ctrl
Inclusion	129	52
Exclusion	632	214
Ψ(PSI)	0.1695	0.1955

State2 $\Delta\Psi$ = -0.0260
normalized state $\Delta\Psi$ = 0.2068

State 3	AD	Ctrl
Inclusion	9	173
Exclusion	16	308
Ψ(PSI)	0.3600	0.3597

State3 $\Delta\Psi$ = 0.0003
normalized state $\Delta\Psi$ = -0.0026

Overall	AD	Ctrl
Inclusion	138	225
Exclusion	648	522
Ψ(PSI)	0.1756	0.3012

Overall $\Delta\Psi$ = -0.1256

$$\text{state } x\ \boldsymbol{\Delta\Psi} = \Psi^{AD}_{state\ x} - \Psi^{Ctrl}_{state\ x} \qquad \text{overall } \boldsymbol{\Delta\Psi} = \Psi^{AD} - \Psi^{Ctrl} \qquad \text{normalized state } x\ \boldsymbol{\Delta\Psi} = \frac{\text{state } x\ \boldsymbol{\Delta\Psi}}{\text{overall } \boldsymbol{\Delta\Psi}}$$

Supplementary Fig. 20. The state dPSI($\boldsymbol{\Delta\Psi}$) is defined by the PSI(Ψ) value difference between AD and control per state. The normalized state $\boldsymbol{\Delta\Psi}$ is obtained by normalizing the state $\boldsymbol{\Delta\Psi}$ by the overall $\boldsymbol{\Delta\Psi}$ ($\boldsymbol{\Delta\Psi}$ across all states combined). Thus, each state is assigned a normalized $\boldsymbol{\Delta\Psi}$, for which we select the maximum to eventually get the maximum normalized state $\boldsymbol{\Delta\Psi}$. **a**, An example shows a high maximum normalized $\boldsymbol{\Delta\Psi}$ in excitatory neurons in AD vs. Control. Both state2 and state3 are confirmed states which have a normalized state $\boldsymbol{\Delta\Psi}$>1, which means the state $\boldsymbol{\Delta\Psi}$ is larger than the overall $\boldsymbol{\Delta\Psi}$ (normalized $\boldsymbol{\Delta\Psi}$=state $\boldsymbol{\Delta\Psi}$/overall $\boldsymbol{\Delta\Psi}$). Within the two confirmed states, the state3 shows the max normalized state $\boldsymbol{\Delta\Psi}$ of 2.102. **b**, An example shows a low maximum normalized $\boldsymbol{\Delta\Psi}$ in excitatory neurons in AD vs. Control. None of the two states are confirmed as both of them have a normalized state $\boldsymbol{\Delta\Psi}$<1, which means the state $\boldsymbol{\Delta\Psi}$ is smaller than the overall $\boldsymbol{\Delta\Psi}$ (normalized $\boldsymbol{\Delta\Psi}$=state $\boldsymbol{\Delta\Psi}$/overall $\boldsymbol{\Delta\Psi}$). Within the two states, the state3 shows the max normalized state $\boldsymbol{\Delta\Psi}$ of 0.2068.Normalized.$\boldsymbol{\Delta\Psi}$=state $\boldsymbol{\Delta\Psi}$/overall $\boldsymbol{\Delta\Psi}$.

Supplementary Table 1

Abbreviations	Full term
PFC	Prefrontal Cortex
VIS	Visual Cortex
L2-3 IT	Layer 2-3 Intratelencephalic neurons
L3-5/L6 IT	Layer 2-3 Intratelencephalic neurons
L5 ET	Layer 5 Extratelencephalic neurons
L6 CT/L6b	Layer 6 Corticothalamic / Layer 6b neurons
L5-6 NP	Layer 5-6 near-projecting neurons
PSI (Ψ)	Percent-Spliced-In or percent spliced in index, which indicates the efficiency of splicing a specific exon into the transcript population of a gene.
dPSI ($\Delta\Psi$)	Delta Percent Spliced-In (PSI) values between two conditions.
LOR	Log odds ratio
