# Supplementary material for: Combined single-cell profiling of chromatin–transcriptome and splicing across brain cell types, regions and disease state
Source: Nat Biotechnol. 2025 Jul 22;44(6):976–88. doi: 10.1038/s41587-025-02734-5 (PMC12425497; doi:10.1038/s41587-025-02734-5)
Supplement: Supplementary file 2 — Reporting Summary [file 41587_2025_2734_MOESM2_ESM.pdf]

nature portfolio

Corresponding author(s): Hagen U Tilgner, Li Gan

Last updated by author(s): 05/28/2025

Reporting Summary

Nature Portfolio wishes to improve the reproducibility of the work that we publish. This form provides structure for consistency and transparency in reporting. For further information on Nature Portfolio policies, see our Editorial Policies and the Editorial Policy Checklist.

Statistics

For all statistical analyses, confirm that the following items are present in the figure legend, table legend, main text, or Methods section.

n/a	Confirmed	
☐	☒	The exact sample size (*n*) for each experimental group/condition, given as a discrete number and unit of measurement
☐	☒	A statement on whether measurements were taken from distinct samples or whether the same sample was measured repeatedly
☐	☒	The statistical test(s) used AND whether they are one- or two-sided<br>*Only common tests should be described solely by name; describe more complex techniques in the Methods section.*
☒	☐	A description of all covariates tested
☐	☒	A description of any assumptions or corrections, such as tests of normality and adjustment for multiple comparisons
☐	☒	A full description of the statistical parameters including central tendency (e.g. means) or other basic estimates (e.g. regression coefficient) AND variation (e.g. standard deviation) or associated estimates of uncertainty (e.g. confidence intervals)
☐	☒	For null hypothesis testing, the test statistic (e.g. *F*, *t*, *r*) with confidence intervals, effect sizes, degrees of freedom and *P* value noted<br>*Give P values as exact values whenever suitable.*
☒	☐	For Bayesian analysis, information on the choice of priors and Markov chain Monte Carlo settings
☒	☐	For hierarchical and complex designs, identification of the appropriate level for tests and full reporting of outcomes
☒	☐	Estimates of effect sizes (e.g. Cohen's *d*, Pearson's *r*), indicating how they were calculated

Our web collection on statistics for biologists contains articles on many of the points above.

Software and code

Policy information about availability of computer code

Data collection	The long read dataset was collected by sequencing on PromethION sequencer, the fastq files were derived from Base-calling performed with MinKNOW 20.06 or MinKNOW 23.07. The short reads datasets were collected by sequencing on NovaSeq 6000 System and fastq files were generated by running bcl2fastq v2.20.
Data analysis	The packages employed in this study: scisorATAC, scisorseqr 0.1.5, cellranger-arc 2.0.1, Seurat 4.2.0 , Signac 1.2.1, MACS2, harmony 0.1.1, seqtk 1.3, DoubletFinder 2.0.3, clusterProfiler 4.2.2, bedtools 2.30.0, BEDOPS V2.4.41, BedtoolsR 2.30.0-5, GenomicRanges 1.46.1, ggplot2 3.4.0, Azimuth 0.4.6, rliger 1.0.0, Velocyto 0.17, Multivelo 0.1.3, LASTZ 1.04.15, scCODA 0.1.9.<br><br>The package scisorATAC is available on github (https://github.com/careenfoord/scisorATAC).<br>Other analysis pipelines involved in this paper are available at https://github.com/wenhu0701/ScisorATAC

For manuscripts utilizing custom algorithms or software that are central to the research but not yet described in published literature, software must be made available to editors and reviewers. We strongly encourage code deposition in a community repository (e.g. GitHub). See the Nature Portfolio guidelines for submitting code & software for further information.

Data

Policy information about availability of data

All manuscripts must include a data availability statement. This statement should provide the following information, where applicable:

- Accession codes, unique identifiers, or web links for publicly available datasets
- A description of any restrictions on data availability
- For clinical datasets or third party data, please ensure that the statement adheres to our policy

The human and macaque short-read and long-read datasets used in this study are available at https://www.ncbi.nlm.nih.gov/sra/PRJNA1021558. All the data used to support the findings of this study are provided within the paper and publicly available at: https://www.gencodegenes.org/human, https://ftp.ensembl.org/pub/release-104/gtf/mus_musculus/, https://www.blueprintnhpatlas.org, https://azimuth.hubmapconsortium.org/references/human_motorcortex/, https://compbio.mit.edu/ad_aging_brain/.

Human research participants

Policy information about studies involving human research participants and Sex and Gender in Research.

Reporting on sex and gender	All the human datasets was generated from the frozen human samples supplied by the Center for Neurodegenerative Disease Research and the University of Pennsylvania Alzheimer's Disease Core Center. The gender information was supplied by the brain bank as follows: 9 Alzheimer's disease(AD) samples: 5 males and 4 females. 10 control PFC samples not diagnosed with dementia: 6 males and 4 females. Considering the difficulty in requesting a large collection of biosamples supplied by the brain bank, the gender composition was considered to be balanced within the control or AD group in this study as much as possible to control for the potential bias stems from sex differences.
Population characteristics	5 AD males, 4 AD females, 6 control males and 4 control females. Age range: 61-87. No race information available.
Recruitment	All the human samples used in this study are de-identified post-mortem frozen samples which were supplied by the tissue banks of UPenn ADCC and CNDR. Thus, this study is considered as "non-human subject research".
Ethics oversight	Acquisition of human tissue samples was done according to institutional review board-approved protocol through the Center for Neurodegenerative Disease Research and the University of Pennsylvania Alzheimer's Disease Core Center.

Note that full information on the approval of the study protocol must also be provided in the manuscript.

Field-specific reporting

Please select the one below that is the best fit for your research. If you are not sure, read the appropriate sections before making your selection.

☒ Life sciences ☐ Behavioural & social sciences ☐ Ecological, evolutionary & environmental sciences

For a reference copy of the document with all sections, see nature.com/documents/nr-reporting-summary-flat.pdf

Life sciences study design

All studies must disclose on these points even when the disclosure is negative.

Sample size	No statistical methods were used to pre-determine sample sizes (e.g., cell number in a single-cell experiment) but we aimed for 5000~10000 single nuclei per sample. These numbers are similar to those reported in previous publications (see PMIDs 31435019, 35256815).<br>No statistical methods were applied to pre-determine the number of individuals used in this study because the macaque and human brain samples are limited resource and hard to obtain. Two prefrontal cortex samples and two visual cortex samples were derived from two male macaque individuals. Nineteen human prefrontal cortex samples (10 controls and 9 AD cases) were supplied by the Center for Neurodegenerative Disease Research and the University of Pennsylvania Alzheimer's Disease Core Center. The number of human samples used in this AD study is similar to the previous single cell studies in AD (e.g. PMID: 34239132).
Data exclusions	No data was excluded.
Replication	2 biological replicates were obtained per brain region(PFC and visual cortex) from macaques . For human post-mortem samples, PFC samples of 10 healthy individuals and 9 AD patients were requested from the Upenn tissue bank. QC and results were replicable, which were confirmed by comparing gene, exon, and isoform expression profiles and chromatin accessibility between replicates.
Randomization	The null hypothesis of the macaque's brain region comparison and species comparison between human and macaque PFC were performed with brain regions of wild type/healthy individuals, thus the healthy condition was considered as no impact on alternative splicing or chromatin accessibility patterns. No experimental manipulations were performed. The study design was hence observational (known samples collected from different regions or species) and did not require randomization of experimental or control groups.<br>For the Alzheimer disease analysis in this study, majority of the AD patients were diagnosed in a late stage (Braak = 5 or 6), which could

	represent a relatively similar disease condition within the group. In addition, to control for the potential effect of gender composition differences in AD or control group, we tried to balance the ratio between genders in each group: 9 Alzheimer's disease(AD) PFC samples composed of 5 males and 4 females, 10 control PFC samples composed of 6 males and 4 females.
Blinding	Not available for blinding design as no treatment was applied.

Reporting for specific materials, systems and methods

We require information from authors about some types of materials, experimental systems and methods used in many studies. Here, indicate whether each material, system or method listed is relevant to your study. If you are not sure if a list item applies to your research, read the appropriate section before selecting a response.

Materials & experimental systems

n/a	Involved in the study
☒	☐ Antibodies
☒	☐ Eukaryotic cell lines
☒	☐ Palaeontology and archaeology
☐	☒ Animals and other organisms
☒	☐ Clinical data
☒	☐ Dual use research of concern

Methods

n/a	Involved in the study
☒	☐ ChIP-seq
☒	☐ Flow cytometry
☒	☐ MRI-based neuroimaging

Animals and other research organisms

Policy information about studies involving animals; ARRIVE guidelines recommended for reporting animal research, and Sex and Gender in Research

Laboratory animals	Two adult male rhesus macaques (26 and 29 years of age).
Wild animals	The study doesn't involve wild animals.
Reporting on sex	The findings only apply to males and no gender related analysis was performed in this study.
Field-collected samples	The study doesn't involve filed-collected samples.
Ethics oversight	All experiments were conducted in accordance with the 2011 Eighth Edition of the NIH guide for the Care and Use of Laboratory Animals. Animal procedures were performed according to protocols approved by the Animal Care and Use Committee of the Rockefeller University.

Note that full information on the approval of the study protocol must also be provided in the manuscript.
